# Chromatin accessibility: biological functions, molecular mechanisms and therapeutic application

**DOI:** 10.1038/s41392-024-02030-9

**Published:** 2024-12-04

**Authors:** Yang Chen, Rui Liang, Yong Li, Lingli Jiang, Di Ma, Qing Luo, Guanbin Song

**Affiliations:** 1 Key Laboratory of Biorheological Science and Technology, Ministry of Education, College of Bioengineering, Chongqing University, Chongqing, PR China; 2 Hepatobiliary Pancreatic Surgery, Yunnan Cancer Hospital, The Third Affiliated Hospital of Kunming Medical University, Kunming, PR China

**Keywords:** Epigenetics, Cell biology

## Abstract

The dynamic regulation of chromatin accessibility is one of the prominent characteristics of eukaryotic genome. The inaccessible regions are mainly located in heterochromatin, which is multilevel compressed and access restricted. The remaining accessible loci are generally located in the euchromatin, which have less nucleosome occupancy and higher regulatory activity. The opening of chromatin is the most important prerequisite for DNA transcription, replication, and damage repair, which is regulated by genetic, epigenetic, environmental, and other factors, playing a vital role in multiple biological progresses. Currently, based on the susceptibility difference of occupied or free DNA to enzymatic cleavage, solubility, methylation, and transposition, there are many methods to detect chromatin accessibility both in bulk and single-cell level. Through combining with high-throughput sequencing, the genome-wide chromatin accessibility landscape of many tissues and cells types also have been constructed. The chromatin accessibility feature is distinct in different tissues and biological states. Research on the regulation network of chromatin accessibility is crucial for uncovering the secret of various biological processes. In this review, we comprehensively introduced the major functions and mechanisms of chromatin accessibility variation in different physiological and pathological processes, meanwhile, the targeted therapies based on chromatin dynamics regulation are also summarized.

## Introduction

**Fig. 1:**
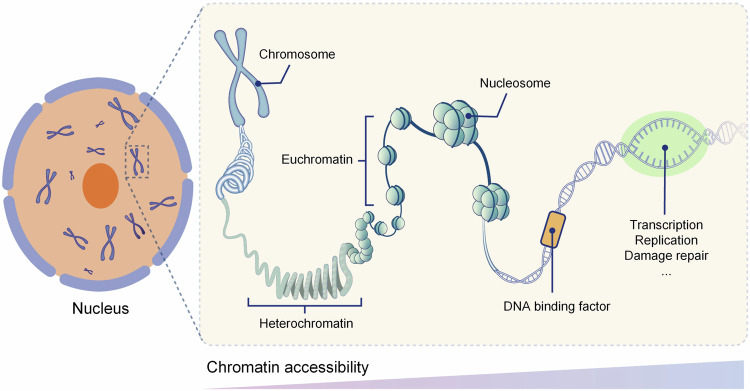
Dynamics of chromatin accessibility in eukaryote. The chromatin of eukaryote is multistage compressed and encapsulated in mitotic interphase nuclei. Majority of the chromatin is highly condensed, which is usually inaccessible. The remaining region is dynamically bound by histones, transcription factors, and other chromatin interaction molecules, characterizing by dynamic accessible, which is crucial for DNA transcription, replication, damage repair, and so on. This picture was drawn by Freescience

Chromatin, a linear complex containing the genetic material, is composed of DNA, histone, non-histone protein, and a small amount of RNA, encapsulated in the interphase nucleus of eukaryote. It can be divided into euchromatin and heterochromatin according to the compression degree. Nucleosome is the basic structural element of chromatin, containing an octamer histone core (two molecules of H2A, H2B, H3, and H4, encircled by ~147 bp of DNA) and a linker histone (H1).^1^ The nucleosomes are densely arranged in facultative and constitutive heterochromatin, while depleted at active regions, such as enhancers, promoters, transcribed gene bodies, DNA replication loci and damage repair sites.^2,3^ Chromatin accessibility refers to the physical contact permissibility of nuclear macromolecules with chromatinized DNA, which is mainly determined by distribution and occupancy of nucleosomes, as well as other DNA-binding factors.^4–6^ The accessible regions only comprise ~2–3% of the whole genome and more than 90% of these regions are yet captured by transcription factors (TFs). The accessibility of specific locus usually reflects its regulatory capacity^3^ (Fig. 1).

The orderly gene expression, DNA replication and damage repair play an important role in maintaining organism homeostasis, and aberrant of which usually results in a variety of diseases, such as cardiovascular diseases, liver diseases, nervous system disorders, diabetes, and neoplasms.^7^ At the different stages of physiological and pathological processes, the gene expression profiles are altered accordingly, which are strictly regulated by TFs.^8,9^ Generally, the TF-mediated gene expression, the orderly DNA replication and damage repair require an appropriate openness state of chromatin.^3,10,11^ Hence, chromatin accessibility plays a critical role in multiple biological processes. The chromatin accessibility is determined by multiple regulatory factors and targeting the chromatin dynamics has obtained many research achievements and improved the treatment of some diseases.

Acquainting the history and actuality of chromatin accessibility research can provide great guidance and help for further investigation. Therefore, in this review, we will introduce the history of chromatin accessibility investigation, common research methods, major mechanisms involved in chromatin accessibility regulation, their functions in different physiological and pathological processes, and targeting therapy strategies in human.

## History of chromatin accessibility research

**Fig. 2:**
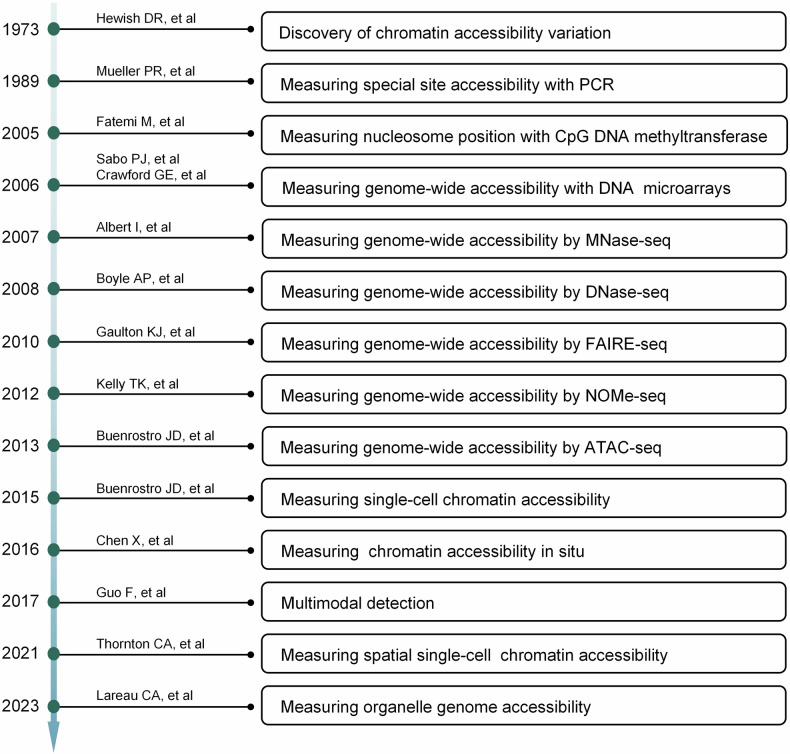
The key discovery in chromatin accessibility research. The research on the accessibility of chromatin can trace back to 1973. Following, many achievements have been obtained in this field. From detecting general accessibility alteration, specific loci changes, to the genome-wide accessibility state. Recently, we are able to detect spatial chromatin accessibility, organelle chromatin accessibility, as well as different epigenetic modifications, transcriptome, and chromatin state in the same specimen at the single-cell resolution. This picture was drawn by Freescience

**Fig. 3:**
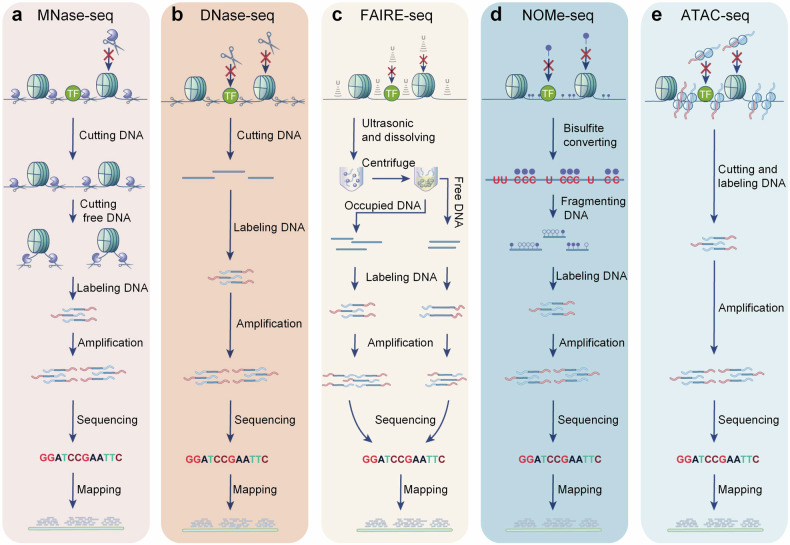
The principal methods to measure chromatin accessibility. The free and occupied DNA are distinct in hypersensitive to MNase- and DNase-mediated cleavage, solubility in different solvents, DNA methylation, as well as transposase-mediated transposition. Combining with high-throughput sequencing, MNase-seq (**a**), DNase-seq (**b**), FAIRE-seq (**c**), NOMe-seq (**d**), and ATAC-seq (**e**) are widely used in genome-wide accessibility studies. This picture was drawn by Freescience

The history of chromatin accessibility investigation is more than five decades. (Fig. 2) The occupied DNA is insusceptibility to enzymatic cleavage, methylation, transposition, and has distinct solubility, which can be applied to investigate the chromatin accessibility. In 1973, Hewish and colleagues found that digesting the nuclear DNA in situ by endonucleases formed conservative periodic stripes, while the free DNA couldn’t. The following work revealed that the DNase hypersensitivity sites (DHSs) within genome were mainly determined by nucleosome distribution.^12–15^ With the invention of polymerase chain reaction (PCR) technology, a series of quantitative methods are applied to measure site-specific chromatin accessibility.^16,17^ In 2005, the nucleosome positions in the promoter of human p16 gene were clarified based on the CpG DNA methylation footprinting.^18^ In 2006, two research groups first measured the genome-wide DHSs using tiled DNA microarrays.^19,20^ In 2007, Giresi et al. invented FAIRE (formaldehyde-assisted isolation of regulatory elements) to detect the chromatin accessibility.^21^ Through combining with next-generation sequencing, many high-throughput methods are applied to research the genome-wide chromatin accessibility, such as MNase-seq,^22^ DNase-seq,^23^ FAIRE-seq,^24^ and NOMe-seq.^25^ Tn5 is a hyperactive transposase that can simultaneously fragment and tag the open genome with designated sequencing adapters. Based on this, Buenrostro et al. invented ATAC-seq (transposase-accessible chromatin using sequencing) in 2013 which simplified and flourished the investigation of chromatin accessibility.^26^ (Fig. 3) Importantly, ATAC-seq has significant advantage to detect the chromatin accessibility of few cells or single cell.^27^

**Fig. 4:**
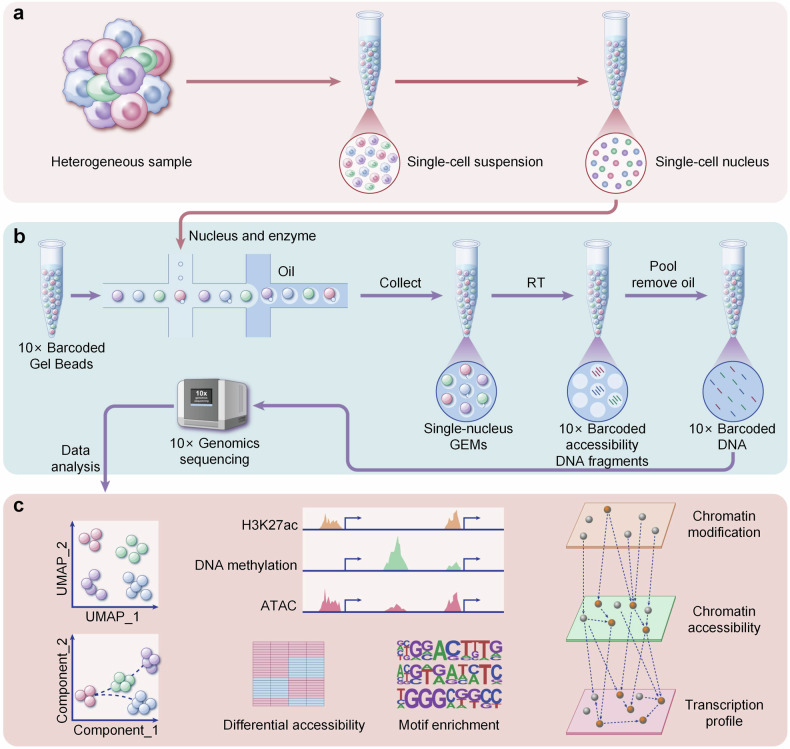
A general process for multimodal single-cell detection of chromatin dynamics. Currently, 10× Genomics sequencing platform is widely applied to obtain the gene expression and epigenetic information in single cell. The process is divided into single-cell nucleus preparation (**a**), library construction and sequencing (**b**), and data analysis (**c**). The sample sources include various tissues or cultured cells. After obtaining single cell suspension, the intact nuclei were obtained by gentle lysis and centrifugation. By adjusting the combination of enzymes, we can obtain gene expression, DNA methylation, histone modification, and chromatin accessibility profiles in the same single cell. Based on the significantly different genes, we can divide cells into different clusters and construct the pseudotemporal differentiation paths, as well as analyze the correlation and difference between gene expression, epigenetic modifications, and chromatin accessibility. This picture was drawn by Freescience

At present, researchers can detect the chromatin accessibility of single-cell,^28^ intractable tissue samples,^29^ single-cell in intractable tissue samples,^30^ and organelle genome.^31^ Particularly, the emergence of multimodal detection techniques makes it possible to directly detect the epigenetic modifications, chromatin states, and gene expression in the same sample at single-cell resolution.^32–34^ By changing the sequencing strategy, it’s also possible to detect the chromatin states and interactions in the relatively long genomic regions.^35,36^ What’s more, uncovering the property of the spatial heterogeneity is very meaningful for the understanding of life and the treatment of diseases. In 2016, Chen et al. detected the chromatin accessibility in situ.^37^ In 2021, Thornton et al. detected the spatial chromatin accessibility at the single-cell resolution.^38^ Recently, many novel strategies have been reported to detect the spatial chromatin accessibility.^39,40^ In summary, new techniques are emerging which make it possible to investigate chromatin accessibility in a faster, more precise, larger scale, higher throughput, and lower cost manner. Meanwhile, the standardization of operation and data analysis processing has brought much convenience to the research.^41,42^ Here, we presented a general process for multimodal detection based on 10× Genomics single-cell sequencing platform (Fig. 4).

## Regulation of chromatin accessibility

The accessibility of chromatin is determined by nucleosome distribution, histone modification, DNA methylation, non-histone protein occupancy, non-coding RNAs, chromatin 3D structure, and so on. In this section, we will introduce the main mechanisms that regulate the chromatin accessibility.

### Nucleosome remodeling regulates chromatin accessibility

**Fig. 5:**
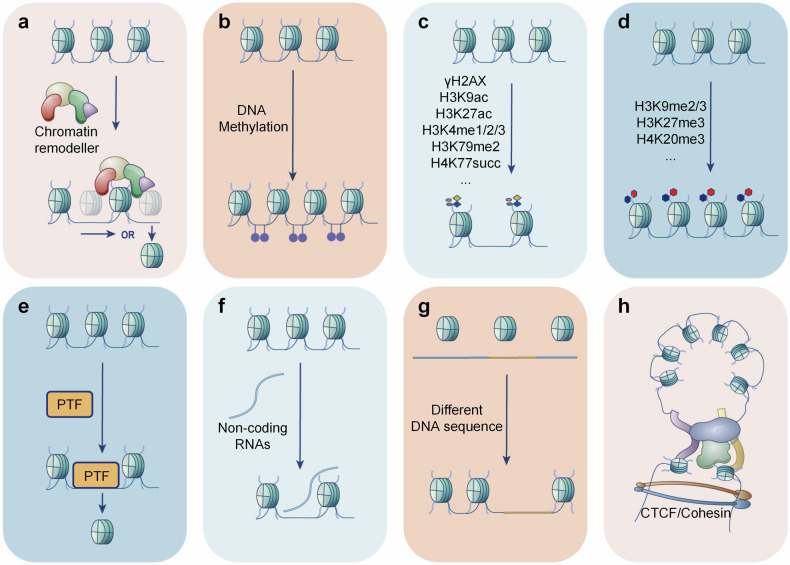
The primary remodellers of chromatin accessibility. The chromatin accessibility is determined by multiple regulators, mainly including chromatin remodellers (**a**), DNA methylation (**b**), histone modifications (**c**, **d**), pioneer transcription factors (**e**), non-coding RNAs (**f**), DNA sequence (**g**), and chromatin 3D structure (**h**). This picture was drawn by Freescience

As the basic chromatin structure, nucleosome positioning is closely associated with chromatin accessibility. ATP-dependent chromatin remodeling complexes are master regulators of nucleosome mobilization. They can switch the “close” or “‘open” state of chromatin by hydrolyzing ATP as energy. According to the distinctive core domains, they are divided into four families, including switch/sucrose-non-fermenting (SWI/SNF), nucleosome remodeling and deacetylation (NuRD), imitation switch (ISWI), and INO80 family.^11^ In this part, we will introduce their mechanisms involved in chromatin accessibility regulation (Fig. 5a).

#### SWI/SNF family

The SWI/SNF complex is the most intensively investigated nucleosome remodeller. This family contains at least 15 subunits, which are divided into BRG1/BRM-associated factor complex (BAF), polybromo-associated BAF complex (PBAF), and non-canonical BAF (ncBAF), depending on different assembly. They can promote or suppress gene transcription by binding with different cofactors.^43^ In 1997, researchers purified partial SWI/SNF complex from rat live tissue and HeLa cells. They found that glucocorticoid receptor (GR) activated SWI/SNF complex to regulate nucleosome disruption in a glucocorticoid response element (GRE) dependent manner. The remolded nucleosome occupancy facilitated the entrance of nuclear factor 1 (NF1) to its target sites, but had no influence on the binding of GR to GREs.^44^ BRM and BRG1 (also called SMARCA2 and SMARCA4, respectively) are the core subunits of SWI/SNF complexes that contain ATPase activity. SMARCA2 and SMARCA4 have distinct effect on the promoter accessibility. In the liver of cholic acid feeding mouse, when treated with farnesoid X receptor (FXR) agonists, SMARCA4 interacted with FXR which subsequently increased the promoter accessibility and transcription of small heterodimer partner (SHP), meanwhile SMARCA2 interacted with SHP to reduce the promoter accessibility and transcription of cytochrome P450 family 7 subfamily A member 1 (CYP7A1) and SHP.^45^ Hemogen can promote the entrance of GATA1/LDB1 complex to its binding motifs by recruiting SMARCA4 while expelling NuRD complex.^46^ Garry et al. revealed that the histone reader PHF7 recruited SWI/SNF complex to the cardiac super enhancers by directly binding with SMARCD3 to facilitate the chromatin opening and expression of target genes in fibroblasts.^47^ AT-rich interaction domain 1A (ARID1A) is another highly conserved subunit of SWI/SNF complex, containing DNA binding ability.^48^ In regenerating liver, lack of ARID1A remolded the histone modification (H3k4me2 and H3k27ac) and decreased chromatin accessibility, which blocked the entrance of hepatocyte nuclear factor 4α (HNF4α), CCAAT enhancer binding protein α (C/EBPα), forkhead box A2 (FoxA2), and E2F transcription factor 4 (E2F4), to their target genes.^49^ Its deficiency also promoted the expression of many cancer stem cell (CSC)-like markers.^50^ In human liver cancer, most of the SWI/SNF members are upregulated. SMARCD1, which increased most significantly, can activate the mechanistic target of rapamycin kinase (mTOR) signaling pathway.^51^ Meanwhile, the mTOR complex 1 (mTORC1) can remodel the chromatin accessibility by promoting ubiquitination-dependent degradation of ARID1A.^52^

#### NuRD family

The NuRD family, also called Mi-2/CHD (Chromodomain, Helicase, DNA binding) complex, contains both histone deacetylase and ATP-dependent chromatin remodeling activities. They can promote transcription by remodeling nucleosomes or suppress that by driving histone deacetylation.^53^ Matsui et al. indicated that FoxAs and PRDM1 recruited NuRD to maintain an accessible nucleosome state during human endoderm differentiation.^54^ During somatic reprogramming, NuRD interacted with Sall4 to reduce the chromatin accessibility of anti-reprogramming genes.^55^ The core ATPase-containing subunits of NuRD complexes include CHD3, CHD4, and CHD5.^56,57^ During B lymphopoiesis, CHD4 reduced the accessibility and expression of many non-B cell lineage genes to convoy the B lymphopoiesis.^58^ In rhabdomyosarcoma, CHD4-containing NuRD complex located to the super-enhancers, establishing a permissive chromatin architecture for the entrance of the tumor driving fusion protein PAX3-FoxO1.^59^ Besides, Shi et al. revealed that CHD4, interacting with SMYD1, suppressed the chromatin accessibility and expression of glycolysis-, hypoxia-, and angiogenesis-related genes during heart development.^60^ Depletion of CHD4 resulted in a globally increased DNA accessibility and induced spontaneous DNA damage in Ewing sarcoma.^61^ RBBP4/7, HDAC1/2, and MTA3 also are important subunits of NuRD complexes. Zhang et al. demonstrated that inhibiting HDAC2 reduced the chromatin accessibility of HDAC2/NuRD binding motifs in HDAC1-deficient neuroblastoma.^62^ Price et al. revealed that DLX1 recruited NuRD by interacting with RBBP4/7 to reduce the chromatin accessibility and expression of Olig2 during subpallium development.^63^ Chanda et al. indicated that NO synthase-induced NO promoted the S-nitrosylation of MTA3 to decrease NuRD activity. Inhibiting NO synthase reduced DNA accessibility in induced pluripotent stem cells (iPSCs).^64^

#### ISWI family

There are at least 16 different ISWI remodellers assembling with diverse subunits. Generally, the ISWI remodellers relocate the nucleosomes, instead of evicting them.^65,66^ In human, SNF2L and SNF2H (also called SMARCA1 and SMARCA5, respectively) are the core subunits of the ISWI family that contain ATPase activity. Goodwin et al. indicated that inactivation of SMARCA1 increased the accessibility of Fos/Jun binding motifs at the promoter regions to activate the ERK signaling pathway in cerebellar granule neuron precursor.^67^ Jiang et al. revealed that the phosphorylated 40S ribosomal protein SA (RPSA) recruited SMARCA5 to increase the chromatin accessibility and expression of NF-κB-targeted genes.^68^ Additionally, the ISWI complex also regulates the DNA repair progress by remodeling chromatin accessibility.^69^

#### INO80 family

The INO80 family contain INO80 (Inositol requiring 80) and SWR1 (SWI2/SNF2-related 1) complexes. Cai et al. found that INO80, interacting with Yin-Yang 1 (YY1), increased the accessibility of YY1 binding motifs and facilitated the YY1-induced transcription.^70^ Ren et al. demonstrated that overexpression of INO80 remodeled the nucleosome landscape and TF binding sites accessibility of the cardiac genes in cardiomyocyte.^71^ In non-small-cell lung cancer (NSCLC), upregulated INO80 increased the genome accessibility and expression of lung cancer-associated genes.^72^ Additionally, HELLS (also called SMARCA6, a member of the SWI2/SNF2 family) increased the nucleosome occupancy of multiple tumor suppressors in hepatocellular carcinoma (HCC) cells.^73^ Besides, INO80 and SWR1 remodeled H2A-H2B assembly in the transcriptional start site (TSS) to regulate gene transcription.^74,75^ The INO80 complexes also ensured the DNA damage repair by maintaining the chromatin accessibility in the damaged loci.^76^

### DNA methylation regulates chromatin accessibility

DNA methylation also participates in the regulation of chromatin accessibility, which always acts as an obstruction for gene transcription. In this part, we will introduce the DNA methylation-mediated chromatin accessibility variation (Fig. 5b).

The DNA methylation is dynamically regulated by methyltransferase and demethylase. DNA (cytosine-5)-methyltransferase (DNMT) and enhancer of zeste 2 polycomb repressive complex 2 subunit (EZH2) are methyltransferases. Inhibition of DNMT and EZH2 widely reduced DNA methylation and increased the chromatin accessibility in HCC cells.^77^ During liver-to-pancreas transdifferentiation, the accessibility of the pancreatic TFs binding motifs was increased, corresponding with reduced DNA methylation. Knockdown of DNMT1 promoted liver-to-pancreas transdifferentiation by increasing the expression of pancreatic-specific genes.^78^ Huang et al. identified a round of DNA demethylation and increasing of chromatin accessibility at meiosis initiation during human spermatogenesis. They confirmed that the reduced expression of DNMT chaperone ubiquitin-like, containing PHD and RING finger domains 1 (UHRF1) contributed to the variation.^79^ Guo et al. also indicated that UHRF1 recruited to the promoter of miR-26b to increase its DNA methylation, leading to reduced chromatin accessibility and miR-26b expression in abdominal aortic aneurysm.^80^

Tet methylcytosine dioxygenase 1 (TET1) is a demethylase. It interacted with TEA domain TF 1/4 (TEAD1/4) to enhance the regional DNA demethylation and H3K27ac of Yes1-associated transcriptional regulator (YAP)-derived genes.^81^ Deng et al. found that RNA m^6^A modification were inversely correlated with DNA 5mC modification both in normal and cancer cells. When binding with m^6^A-modified RNA, reader protein FXR1 recruited TET1 to the transcriptional loci to demethylate DNA, resulting in increased chromatin accessibility and gene transcription.^82^ 5-hydroxymethylcytosine (5hmC) is an active DNA modification that modified by TETs. Li et al. indicated that the 5hmC level is dynamically regulated during pancreatic differentiation of human embryonic stem cells (hESCs) which corresponded with chromatin accessibility and gene transcription activity.^83^

The plasma cell-free DNA (cfDNA) exists in human peripheral blood. Studies indicated that low level of DNA methylation also increased nucleosome accessibility of cfDNA which was in line with nuclease sensibility, cutting site, and size distribution.^84,85^ Perinatal expression of deiodinase 2 (D2) also remolded the gene expression and DNA methylation pattern in adult mouse hepatocytes. D2 insufficiently reduced the overall chromatin accessibility which was in line with increased DNA methylation, but the modification enzyme is unclear.^86^

### Histone modification regulates chromatin accessibility

Histones are the basic structural proteins of chromosome, rich in alkaline amino acid (arginine and lysine), which can facilitate the binding of acid DNA. Up to now, a great number of histone post-translation modifications (PTMs) are confirmed, such as phosphorylation,^87^ methylation,^88^ ubiquitylation,^89^ acetylation,^90^ lactylation,^91^ and crotonylation,^92^ which make up the histone code. The PTM landscapes of histones are closely related to the accessible state of chromatin. In this part, we will introduce the chromatin accessibility variant determined by histone modification. (Fig. 5c, d)

#### Histone phosphorylation

Phosphorylation is one of the most common and important PTMs in histones. The histones are phosphorylated and dephosphorylated periodically corresponding with the condensation and unwrapping of chromatin during mitosis, which indicates that histone phosphorylation is a crucial regulator of chromatin dynamics.

It’s well confirmed that phosphorylation of H3 at different serine or threonine residues can increase the accessibility of chromatin to facilitate gene transcription and DNA repair.^93,94^ Covalently closed circular DNA (cccDNA) is the hereditary material of the hepatitis B virus (HBV), which forms a microchromosomes in the hepatocyte nuclei. Ming et al. indicated that high mobility group nucleosome binding domain 1 (HMGN1) promoted the accessibility and transcription of cccDNA by competitively combining with H1 and promoting H3 phosphorylation.^95^ The H3 phosphorylation also orchestrates other PTMs, such as H3K9me3 and H3K36me3.^96^

Phosphorylation of H2AX is an important indicator for DNA damage repair. Researches indicated that the phosphorylated H2AX, called γH2AX, specifically recruited to the DNA double strand break sites, maintain the accessible of damaged DNA to facilitate the following repair pathway.^97^ H2A.Z is another variant of H2A. Fuglerud et al. indicated SRY-box transcription factor 9 (Sox9)-induced open chromatin was main located in H2A.Z enriched regions, and the H3S28 phosphorylation prevent the binding of Sox9 with chromatin.^98^

Histone H1 is the linker histone, regulating the higher-ordered chromatin structure. Researches indicate that the expression level of H1 is closely related to the chromatin structure.^99^ Meanwhile, the phosphorylation of H1 regulates its affinity to chromatin.^100^ The ex vivo research indicated that partial phosphorylation of H1 increased chromatin accessibility.^101^ However, in vivo evidence for the direct relationship between H1 phosphorylation and chromatin accessibility is still lacking.

#### Histone methylation

Histone methylation, including monomethylation, dimethylation, and trimethylation, usually occur on the lysine or arginine residues at the N-terminus of H3 and H4. Their role on chromatin structure and gene expression are site- and quantity-dependent.^102^

Kochat et al. indicated that downregulation of the methyltransferase EZH2 reduced H3K27me3 modification of many hepatic development-related genes, promoting the reprogramming of bone marrow progenitor cells to hepatocytes.^103^ Boonsanay et al. confirmed that loss of the methyltransferase SUV420H2 reduced H4K20me3 modification and increased chromatin accessibility predominantly in colorectal cancer organoids.^104^ Disruptor of telomeric silencing 1-like (DOT1L) is the only known methyltransferase catalyzing the methylation of H3K79.^105^ Researches indicated that DOT1L-induced H3K79me2/3 modification was important to maintain the accessible state of chromatin both in MLL-AF4 and mouse embryonic stem cells.^106,107^ Yang et al. indicated that the H3K4-specific methyltransferase MLL2 promoted the H3K4me modification to increase the accessibility of GR-targeted genes in ARPE-19 cells.^108^ PRMT5 is an arginine methyltransferase. Dacwag et al. revealed that PRMT5 catalyzed H3R8me2 modification, facilitating the binding of SWI/SNF complex with the promoter of target genes and enhancing their accessibility.^109^ Recent research indicated that SMARCA4 facilitated the binding of PRMT5 with the promoter of FoxO1 to maintain its H3R2 methylation and accessible state.^110^ These researches indicated that PRMT5 and SWI/SNF complex may cooperate to maintain the chromatin accessibility.

The JmjC domain-containing family (JMJDs) is a histone demethylase superfamily. In the same research, Kochat et al. they demonstrated that the upregulated JMJD3 was accountable for the reduction of H3K27me3 modification.^103^ Synergistically utilization of donafenib (multi-kinase inhibitor) and GSK-J4 (JMJD3 inhibitor) increased the promoter accessibility and expression of heme oxygenase 1 (HMOX1) by reducing H3K27me3 modification and simultaneously enhancing H3K4me1 and H3K27ac modifications.^111^ Additionally, JMJD1C enhanced the promoter accessibility of lipogenic genes by reducing the H3K9me3 modification.^112^ Lysine demethylase 1A (KDM1A) and KDM5B are histone demethylases. KDM1A exacerbated metabolic dysfunction-associated steatotic liver disease (MASLD) by increasing chromatin accessibility.^113^ KDM5B inhibited Nfkbia expression by reducing its promoter H3K4me3 modification and accessibility.^114^ Zhang et al. demonstrated that KDM4 (JMJD2) reduced the H3K9me2/3 and H3K36me2/3 modification to maintain the accessibility and expression of aging-related genes in prostate cancer cells.^115^

#### Histone ubiquitination

Ubiquitination is an universal modification to determine the protein stability, location, activation, and interaction. The ubiquitination of histone can remodel the chromatin structure, transcript elongation, and DNA damage repair.

Segala et al. revealed that the E3 ligase complex RNF20/RNF40 catalyzed the monoubiquitylation of histone H2B (H2Bub1) to block the INO80-mediated eviction of H2A.Z in the inducible enhancer regions and reduce their accessibility.^116^ Hooda et al. indicated that RNF20 insufficiency-induced H2Bub1 loss promoted the chromatin accessibility and expression of immune signaling pathways.^117^ Lin et al. also indicated that RNF20 increased the H2Bub1 to enhance the promoter accessibility and expression of many genes.^118^ Yin et al. indicated that the H2AK121ub modification was located in the less accessible but still permissive chromatin regions at transcriptional regulation locus.^119^ The loss of deubiquitinating enzyme BAP1 promoted H2AK119ub modification, remodeling the chromatin accessibility to perturb the transcriptomic pattern in human ductal liver organoids.^120^ Zhang et al. revealed that the H2BK120ub modification was important for the maintenance of accessible chromatin fiber to facilitate DNA repair.^121^ Additionally, the unbiquitination of histones always synergy with other modifications. Huang et al. demonstrated that the H2BK123ub inhibited Jhd2-induced H3K4 demethylation to maintain chromatin accessibility.^122^ Similarly, Worden et al. indicated that H2BK123ub facilitated DOT1L-induced histone H3K79me to increase the chromatin accessibility.^123^

At present, the research on histone ubiquitination and chromatin accessibility mainly focuses on H2B and H2A. The ubiquitination modification on other histones and their functions on chromatin accessibility still need to be investigated.

#### Histone acylation

Lysine acetylation is the most common acylation modification types on histones. Acetylation reduces the positive charge of histones to weaken their DNA binding capacity and inhibit the formation of higher chromatin structures, usually corresponding with higher accessibility and transcription activity.

Multiple acetylation modification sites have been clarified in different histones, which are dynamically regulated by acetyltransferase and deacetylases.^124^ In transforming growth factor β (TGFβ) treated cholangiocytes, SMAD family members recruited the histone acetyltransferase KAT2A to increase the H3K9ac modification and chromatin accessibility in the promoters of hematopoietic stem cell (HSC)-activating genes.^125^ Mutation of KAT6A reduced the H3K23ac modification and chromatin accessibility of HOXC gene cluster in dermal fibroblasts.^126^ Muthukrishnan, et al. confirmed that the histone acetyltransferase P300 increased the H3K27ac modification and chromatin accessibility of specific genes in glioma CSCs.^127^ The TFs c-Jun and Klf5 can recruit CBP/P300 to the promoter of special genes, which increases their H3K27ac modification, accessibility, and expression.^128,129^ Samata et al. indicated that H4K16ac was essential to maintain chromatin accessibility for the zygotic genome activation.^130^

The acetylation on histones can be eliminated by histone deacetylases. In the progression of metabolic steatohepatitis (MASH), methyltransferase-like 3 (METTL3) interacted with HDAC1/2 to remove the H3K9ac and H3K27ac modification in the promoters of CD36 and C-C motif chemokine ligand 2 (CCL2).^131^ HDAC8 reduced H3K27ac modification at the enhancers of CCL4 in HCC cells.^132^ SPI1 recruits HDAC1 to the active enhancers which globally reduces the promoter acetylation, chromatin accessibility, and RNA pol II occupancy in leukemic cells.^133^ Nucleosome assembly protein 1-like 2 (NAP1L2) recruits the deacetylase SIRT1 to evict H3K14ac modification on promoters of osteogenic genes and reduce their accessibility and expression in bone marrow mesenchymal stem cells (BMSCs).^134^ P53 recruits SIRT1 to reduce the H3K27ac modification, promoter accessibility, and transcription of Neat1.^135^ Yuan et al. indicated that ZCWPW1 inhibited HDAC-induced H3K9 deacetylation to maintain the openness of chromatin during meiotic double-strand break repair.^136^

The upregulation of acetyl-CoA metabolism can provide more substrate for acetylation. Liu et al. revealed that vitamin B1 increased the histone acetylation and chromatin accessibility by facilitating acetyl-CoA metabolism.^137^ Pyruvate dehydrogenase 1α (PDHE1α) drives acetyl-CoA production by catalyzing pyruvate. It increases the acetyl-CoA level in the DNA damage sites to facilitate histone acetylation and chromatin accessibility.^138^

Recently, many novel acylation modifications on histone are constantly identified which participate into the regulation of chromatin dynamics. In 2019, Zhao group identified lactylation on histones which can remodel the expression atlas in macrophage.^139^ Recently, Merkuri et al. demonstrated that glycolysis induced the histone lactylation at neural crest related genes, which increased their chromatin accessibility and expression.^140^ Trujillo et al. also indicated that lactylation of histone increased the chromatin accessibility to promote inflammatory signaling in macrophages.^141^ Jing et al. demonstrated that, succinylation of H4K77 (H4K77succ) promoted DNA unwrapping from the histone surface, facilitating the binding of proteins with the nucleosome DNA.^142^ Additionally, serotonylation has been confirmed to exclude from constitutive heterochromatic regions which hints that serotonylation is a potential active marker for chromatin.^143^

In addition, other factors also regulate chromatin accessibility by remolding the histone modification profiles. For example, the HBV X protein (HBx) increases the H3K27ac modification and super enhancer accessibility of ETS variant TF 4 (ETV4), thereby promotes its expression.^144^ Interleukin 6 (IL-6) and tumor necrosis factor α (TNF-α) treatment increase the H3K4me3 modification and promoter accessibility of microtubule-associated serine/threonine kinase like (MASTL).^145^ Mitochondrial stress increases the H3K4me1 and H3K27ac modifications as well as the chromatin accessibility in amphiregulin (AREG) enhancer regions.^146^ Knockdown of nuclear autoantigenic sperm protein (NASP) decreases H3K9me1 modification and enhances chromatin accessibility globally.^147^ Sox4 modifies the landscapes of H3K27ac, H3K4me1, and H3K4me3 modifications during the hepatocytes to biliary transdifferentiation.^148^ ARID1A deletion decreases H3K4me3 and chromatin accessibility on the promoters of fatty acid oxidation (FAO)-related genes.^149^ The co-transcription factor (co-TF) VGLL1, binds with TEAD4 to increase the accessibility and histone acetylation modification at target gene loci.^150^ Mineral dust-induced gene (MDIG) can promote the H3K9me3-to-H3K9me1 transformation of OTX2 promoter which facilitates the entrance of Myc.^151^ However, these regulators cannot modify histone directly, the direct participant involved in these processes needs further elucidation.

### Pioneer transcription factors regulate chromatin accessibility

Early studies have shown that TFs can enhance chromatin openness.^152^ Now, we know that majority of these TFs are pioneer transcription factors (PTFs). Different from conventional TFs, PTFs can bind with closed chromatin regions and enhance the accessibility of local chromatin which facilitate the entrance of other tissue-specific TFs to control cell fate and function (Fig. 5e).

The members of FoxA family are the most important PTFs in liver. They remold chromatin accessibility to regulate the development and function of liver.^153^ FoxAs can expel linker histone H1, thereby maintaining the accessibility of liver-specific enhancers and promoters to permit the entrance of other liver-specific TFs,^154^ such as HNF4α, FXR, and liver X receptor α (LXRα).^155,156^ Research indicated that FoxA2 binding sites are located in nucleosome-free regions which showed hypersensitive to MNase.^157^ In the adult liver, HNF4α, but not FoxA2, is required for maintaining the chromatin structure.^158^ HNF4α enriched H3K4me1 and H3K27ac modification to maintain opening chromatin at active transcriptional regions.^159^ HNF4α also enhanced the accessibility of basic helix-loop-helix ARNT-like 1 (BMAL1) binding motifs to regulate liver circadian rhythms.^160^ Exogenous expression of Yamanaka factors (Sox2, Oct-3/4, Klf4, and c-Myc) in adult somatic cells can facilitate them dedifferentiating into iPSCs, which involves many epigenetic remodeling.^161,162^ Research indicated that inducible expression of Yamanaka factors promoted the expression of DNA topoisomerase II alpha (TOP2A) to modify the chromatin accessibility.^163^ Fuglerud et al. indicated that Sox9 can unwrap chromatin at special sites in closed chromatin. It promoted the expression of endothelial-to-mesenchymal transition genes by increasing their chromatin accessibility.^98^

Research also revealed that FoxA2 binding alone did not increase the permissive of chromatin. Only synergistically binding with other cofactors can FoxA2 enhance chromatin accessibility.^164^ These results indicate that some PTFs are more likely to play a positioning function. When bind to specific chromatin site, they recruit other chromatin regulators to synergistically regulate the local chromatin accessibility.

### Non-coding RNAs regulate chromatin accessibility

The non-coding RNAs, including microRNAs (miRNAs), long non-coding RNAs (lncRNAs), circular non-coding RNAs (circRNAs), and so on, perform many important biological functions. Researches have confirmed that they are master regulators of chromatin accessibility (Fig. 5f).

MiR-137 is a neuropsychiatric-disorder-associated miRNA that located in the microglial nucleus. Li et al. indicated that miR-137 decreased the chromatin accessibility by competitively binding with microglial master transcription factor Pu.1 to suppress the binding of Pu.1 with chromatin.^165^ Boos et al. indicated that the hypoxia-induced lncRNA LINC00607 was essential for normal endothelial function and angiogenic sprouting. It regulated the chromatin accessibility around the binding motifs of ETS transcription factors ERG, by directly interacting with the chromatin remodeller SMARCA4.^166^ In the earlier research of the same group, they found that SMARCA4 can interact with lncRNA MANTIS, to facilitate the binding of RNA Polymerase II to DNA.^167^ Ma et al. indicated that the lncRNA HOTAIR, increased the chromatin accessibility and expression of metastasis-related genes in breast cancer cells, but the detail mechanism requires to reveal.^168^ Additionally, circRNA circTmem241 recruited methyltransferase Ash1l to the promoter of Elk3 and enhanced its chromatin accessibility and transcription in innate lymphoid cells.^169^ Besides, synthetic RNA increased the chromatin accessibility of albumin gene in vitro but its function in vivo is also unclear.^170^

Dueva et al. indicated that single-stranded RNA in the nucleus maintain an open chromatin structure by interacting with histone tails to neutralize the positive charge on histones.^171^ In 2020, He et al. proposed a concept, chromosome-associated regulatory RNAs (carRNAs), including promoter-associated RNAs, enhancer RNAs, repeat RNAs, and so on, which can regulate the chromatin structure in mammalian cells.^172^ Members of long interspersed element-1 (LINE1) family are representative carRNAs, that have been confirmed to regulate chromatin structure.^173^ He et al. indicated that m^6^A modification decreased the carRNA level, especially LINE1 to reduce the chromatin accessibility and nascent RNA transcription.^172^ Li et al. indicated that the m^6^A modification stabilized the super-enhancer RNAs, which consequently recruited H3K4 methyltransferase MLL1 to promote H3K4me3 modification and accessibility accessibility in specific genes.^174^

### Cis-regulatory element regulates chromatin accessibility

The cis-regulatory elements, which specifically bound by DNA binding proteins (TF, co-TF, transcription inhibitor, transposase, etc) are nonnegligible regulators of chromatin accessibility. In vivo researches have showed that nucleosomes exhibit significant DNA sequence preferences.^1^ Li et al. indicated that mutation of human enhancers altered their accessibility.^175^ The single nucleotide polymorphisms (SNPs) also determine the chromatin accessibility and TF affinity.^176,177^ Spisak et al. edited the SNPs in prostate cancer cells significantly remodeling the chromatin accessibility, histone modification, and TF affinity.^178^ Recently, Mononen et al. demonstrated that the genetically driven differences in the expression pattern, H3K27ac modification, and chromatin accessibility were more pronounced than those induced by diet in mouse liver.^176^ (Fig. 5g)

### The three-dimensional (3D) structure of chromatin regulates its accessibility

The chromatin is orderly and dynamically encapsulated in the nucleus, and the 3D structure plays an important role in regulating its accessibility. (Fig. 5h) The lamin A/C variation-induced morphology change of nucleus is closely related to chromatin accessibility, epigenetic modification, and gene expression.^179^ Besides, the nuclear matrix protein, heterogeneous nuclear ribonucleoprotein U (hnRNPU) has been reported to regulate chromatin accessibility.^180^ Mitochondrial TF A (TFAM) remolded the chromatin accessibility by inducing the polymerization of nuclear actin.^181^

At present, the chromatin conformation capture (3C) technologies allow us to measure the 3D structure of chromatin directly. These 3D structures mainly include compartments,^182^ loop domains,^183^ topologically associated domains (TADs),^184^ and so on. Cohesin and CCCTC-binding factor (CTCF) are the key architectural proteins that regulate the formation of TAD and loop domain.^185^ Xie et al. indicated that Cohesin, CTCF together with BRD2 protected the architectural boundaries of accessible chromatin regions.^186^ Chen et al. demonstrated that the TAD boundaries were more accessible, contained higher transcriptional capacity and more DNA double-strand breaks (DSBs).^187^ Li et al. indicated that the chromatin 3D structure and accessibility determined the pluripotent state of embryonic stem cells (ESCs).^188^ The variation of the chromatin accessibility and higher structure also coincided with the neuron development. Wahl et al. revealed that SATB2 remodeled the chromatin 3D structure and accessibility both independent and in cooperation with CTCF in cortical neurons.^189^ Additionally, many researches have indicated that the feature of chromatin 3D structure and accessibility were distinct in different diseases, such as breast cancer,^190^ glioma,^191^ and Alzheimer’s disease (AD).^192^ It indicates that the chromatin 3D structure could be an potential disease biomarker and therapeutic target.

It’s sure that the higher structure and distribution of chromatin are closely related with chromatin accessibility and gene expression. However, whether the variation of chromatin 3D structure is a cause or consequence of chromatin accessibility change is still controversial,^193^ hence, further investigation on this field is required urgently.

### Environmental factors regulate chromatin accessibility

The surrounding environment is an important regulator of biological process. Many environmental factors can directly regulate gene expression. In this section, we summarized the function of environmental factors on chromatin accessibility.

Chemical exposure-induced epigenetic alteration is attracting more and more attention in human health. The environmental chemicals can widely remold the chromatin accessibility and TF binding patterns. Israel et al. indicated that exposure to genotoxic carcinogen 1,3-butadiene widely remolded the histone acetylation, chromatin accessibility, and gene expression patterns in lung, kidney, and liver tissues of C57BL/6J and CAST/EiJ mice.^194^ Hexavalent chromium (Cr(VI)) is well-clarified respiratory carcinogens by forming protein-Cr-DNA adducts. VonHandorf et al. indicated that Cr(VI) dysregulated the nucleosome occupancy at specific genome locus, blocking the activator protein-1 (AP-1) and CTCF-targeting motifs in mouse liver.^195^ Acrolein is abundant in cigarette smoke. Chen et al. indicated that acrolein exposure increased the chromatin accessibility through compromising the delivery of H3 into chromatin.^196^ Bisphenol F also remodeled the hepatic transcriptome, metabolome, and chromatin accessibility to trigger MASLD.^197^ Ionizing radiation is an ubiquitous environmental pathogenic factor. Dahl et al. found that radiation induced globally chromatin accessibility alteration in mouse liver.^198^ In mouse hepatoma cells, dioxin-induced promoter accessibility of CYP1A1 facilitated the formation of AhR/Arnt heteromer.^199,200^ These studies have suggested that the harmful environmental factor-induced chromatin accessibility alteration played an important role in disease progression. Besides, environmental factor is irreplaceable for the regulation of our daily routine. The related role and mechanism in these progresses are remained to be elucidated.

In addition to mentioned above, many other regulators may also participate in the remolding of chromatin accessibility. For example, folic acid increased the accessibility of IGF2 promoter during embryonic development of broiler.^201^ The mechanical signals of the extracellular matrix also regulates the chromatin accessibility.^202^ However, their variation, manifestation, function, and mechanism in the physiological and pathological processes are required further investigation.

## Chromatin accessibility in physiological processes

**Fig. 6:**
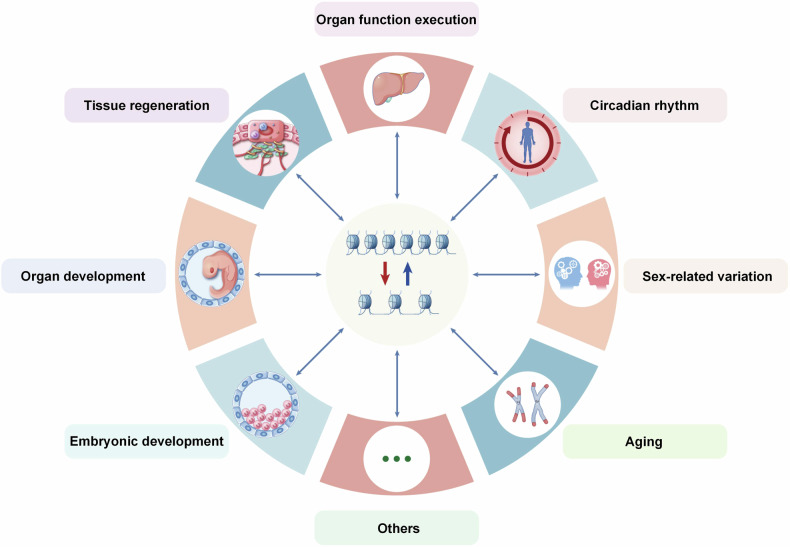
Chromatin accessibility variation is involved in multiple physiological processes. The chromatin accessibility is dynamically varied in many physiological processes, such as embryonic and organ development, tissue regeneration, organ function execution, circadian rhythm, gender difference, senescence, and so on. This picture was drawn by Freescience

In the previous section, we introduced the major molecular mechanisms of chromatin accessibility regulation. The variation of chromatin accessibility participates in many physiological processes, such as early embryogenesis, organ development, tissue regeneration, aging, circadian rhythms, and so on. Researchers have constructed the chromatin accessibility atlas of different human organs and identified the specific regulatory networks in different cells. In this section, we will introduce the detail functions and mechanisms of chromatin accessibility variant in some physiological processes (Fig. 6).

### Chromatin accessibility in early embryonic development

All mammals are originated from the fusion of male and female gametes, called zygote. After fertilization, the embryo is constantly dividing, orderly forming morula, blastocyst, gastrula, and finally differentiated into various tissues and organs. Following fertilization, the highly specialized epigenetic modification of sperm and oocytes are reprogrammed to facilitate the establishment of a totipotent state which is required for the embryo development. The division of human embryo in the first 3 days is mainly sustained by maternally inherited factors, and a major wave of embryonic genome activation (EGA) arises at the 4-cell (4C) to 8-cell (8C) stage.^203^ The epigenetic modification of embryo chromatin changes significantly which is in line with its accessibility variation during embryogenesis. It’s consistent in many researches that the accessibility regions of human embryonic genome are increased progressively from the 2-cell (2C) to blastocyst stage.^204,205^ Most of the accessible regions are located in the promoters, CpG islands, and enhancers. Although some genes containing accessible promoter in the 2C stage do not transcribed until at the 8C stage, their are more highly expressed in the subsequent stages than those gain accessibility later. It indicated that accessibility state poised in the early stage is critical for the high expression of some critical genes.^206^

It is of great significance to clarify the contribution of the remodellers on chromatin accessibility variation during the early embryonic development. Samata et al. demonstrated that the H4K16ac modification was intergenerationally chromatin modification from oocytes to fertilized embryos. It’s indispensable to maintain the chromatin accessibility for zygotic genome activation. Maternal depletion of the acetyltransferase MOF resulted in H4K16ac loss and downregulation of post-zygotically expressed genes.^130^ Treating the 8C embryo with the transcription inhibitor α-amanitin resulted in a similar distal regulatory element accessibility pattern with pre-EGA stage, indicating that transcription is indispensable for chromatin accessibility variation, at least in distal elements at EGA stage.^205^ In human embryos, the direct evidence about the relationship between chromatin accessibility and its remodeller is yet to be revealed, but some leads are promising. The inverse relationship between accessibility and DNA methylation occurs at all stages of human early embryogenesis, and the expression pattern of many gene families with repetitive elements are coincident with their methylation modification and chromatin accessibility during preimplantation development.^204^ Besides, the subunits of SNF2-family regulate Oct-4 in naive human pluripotent stem cells to orchestrate the expression of blastocyst lineage genes.^207^ The H3K9me3 modification landscape is also varied during preimplantation embryo development.^208^ Given that SNF2 complex, DNA hypermethylation, and H3K9me3 are regulators or indicators of chromatin accessibility, they could contribute to the chromatin variation in human early embryogenesis.

### Chromatin accessibility in organ development

The human body consists of many tissues and organs, all of which are derived from the epiblast of the blastocyst. Meanwhile, the maintenance and differentiation of somatic stem cells are also crucial for maintaining organ homeostasis. The determination of cell fate is crucial for the orderly development of different tissues and organs. Multiple researches have indicated that the chromatin accessibility is an important regulator of cell fate. In undifferentiated pluripotent cells, the tissue-specific cis-regulatory elements usually reside in the closed, silent chromatin regions that are hard for TFs entrance. Besides, the chromatin accessibility is distinct in different tissues and organs of mammals. These differences are mainly determined by tissue-specific PTFs, which can bind with heterochromatin and permanently change the epigenetic chromatin modification and stably maintain the accessibility of tissue-specific genes.^209^ In this part, we will discuss the variation and function of chromatin accessibility during different organ development.

#### Heart development

Heart is the first functional organ during embryonic development. The epigenetic modification, which can determine the chromatin accessibility, is a master regulator of cardiac development.

In 2022, Ameen et al. constructed a single-cell resolution chromatin accessibility atlas of human fetal heart tissues. They defined a series of cell types in the heart by different TFs and identified the developmental trajectories of human fetal heart. Meanwhile, by comparing the chromatin accessibility profiles of congenital heart disease (CHD) cases and normal controls, they identified many potential CHD-inducing factors, among which they confirmed that loss of JARID2 impaired heart development.^210^ Researches have indicated that H2Bub modification was critical for cardiac development, and the mutations of E3 ligases RNF20, which regulates H2Bub modification, commonly occur in CHD patients.^211,212^ Recently, Lin et al. indicated that RNF20 increased the promoter accessibility and expression of cell-cell connections and actin organization related genes by monoubiquitylating histone H2B to promote postnatal cardiomyocyte polarization.^118^ The TF FoxK1 is specifically expressed in developing cardiac and skeletal muscles.^213^ It can promote the proliferation of myogenic stem cell following injury.^214^ Sierra-Pagan et al. indicated that FoxK1 also promoted the development of mesodermal progenitor cells by orchestrating the chromatin accessibility of cardiac developmental genes, especially inhibiting the Wnt/β-catenin signal pathway.^215^ GATA4/5/6 are essential and conserved TFs that regulate heart development.^216^ Song et al. indicated that GATA5/6 determined the balance of cardiac and pharyngeal development. GATA5/6 regulated globally chromatin accessibility to orchestrate the expression of cardiac and pharyngeal regulatory genes.^217^ Arrieta, et al. indicated that knockdown the circadian protein BMAL1 in ventricular myocytes impaired the postnatal development of rat heart. The loss of BMAL1 decreased the accessibility of Per2 and Sik1 promoter.^218^ Zhong et al. indicated that knockout c-Jun facilitated the differentiation of hESCs into cardiomyocytes in vitro. C-Jun deficiency increased the chromatin accessibility of hESCs. Mechanically, loss of c-Jun increased the expression of RBBP5 and SETD1B, increasing H3K4me3 deposition on cardiogenesis-related genes.^219^ Krup et al. indicated that knockout Mesp1 impaired the differentiation of cardiac mesoderm cells to cardiomyocytes. ScATAC-seq analysis revealed that Mesp1-KO cells showed strikingly divergent regulatory landscapes compared with controls. Mesp1 insufficiency reduces the promoter accessibility and expression of cardiac differentiation-driving genes.^220^ The TFs Wt1a and Wt1b blocked cardiomyocyte differentiation by reducing the chromatin accessibility of cardiomyocyte-specific genes.^221^ Fang et al. revealed that deletion of TET2/3 decreased the global 5hmC modification and chromatin accessibility, perturbing YY1 binding to disrupt cardiac-specific transcription.^222^ Meier et al. constructed the single-cell transcriptome and chromatin accessibility profiles of human epicardioids. Through combining lineage tracing, they found that epicardioids were derived from first heart and juxtacardiac field progenitors. Additionally, the in vitro treated epicardioids can mimic left ventricular hypertrophy and fibrosis, offering an unique testing ground for heart epicardial development, disease, and regeneration.^223^

Chromatin accessibility variation is also associated with the pacemaker development in the sinoatrial node (SAN).^224^ Expression of GATA4, Tbx5, and Mef2c combining with or without Hand2 can reprogram fibroblasts into induced cardiomyocyte-like myocytes (iCLMs) or pacemaker-like myocytes (iPM), respectively.^225,226^ Fernandez-Perez et al. indicated that Hand2 promoted the expression of pacemaker-specific genes by increasing their promoter accessibility.^227^ Galang et al. compared the accessible chromatin atlas of cardiac pacemaker cells with that of right atrial cardiomyocytes. They found that the accessibility of an Isl1 enhancer was increased in the SAN, which promoted the expression of Isl1, thus facilitating SAN development. By analyzing the ATAC-seq peak around Isl1 enhancer, they speculated that this Isl1 enhancer also regulated SAN development in human.^228^ van Eif et al. analyzed the human accessible chromatin both in pluripotent stem cell-derived SAN-like pacemaker cells (SANLPCs) and ventricle-like cells. They confirmed that the Isl1 locus was more accessible in SANLPCs. Besides, they identified that the Tbx3 enhancer is also enriched. Specific-deletion of the homologous region in mouse model impaired the SAN development.^229^

#### Liver development

**Fig. 7:**
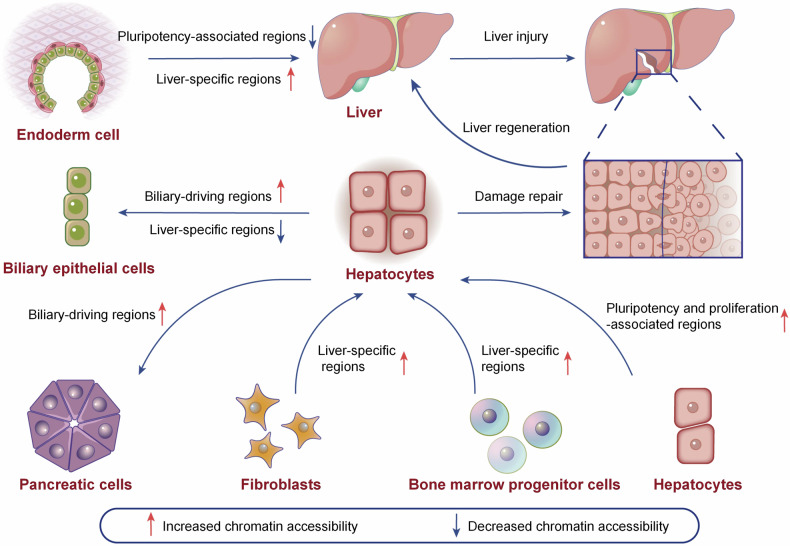
Chromatin accessibility regulates the development, regeneration, and transdifferentiation of liver. During the development of liver, the chromatin accessibility of pluripotent genes and liver-specific genes are reduced and increased, respectively. During the repair of damaged liver, the chromatin accessibility of pluripotency and proliferation-related genes are increased. During the transdifferentiation of liver, the specific gene accessibility of origin tissue is always reduced and that of the aim tissue is correspondingly increased. This picture was drawn by Freescience

The orderly development plays a vital role in maintaining liver homeostasis. During liver development, the chromatin structure, epigenetic modifications, and gene expression profile are changing correspondingly (Fig. 7).

FoxA and GATA families can remold the chromatin structure of liver-specific genes, facilitating the access of hepatic TFs to their target genes.^230,231^ Reizel et al. demonstrated that FoxAs increased the enhancer accessibility of HNF4α-targeting genes to maintain liver homeostasis.^232^ Besides, the DNA methylation profiles are altering during development, manifested as de novo methylated of pluripotency genes and demethylation of tissue-specific genes, which are remarkably consistent with chromatin accessibility and gene expression.^233^ In broiler, folic acid injection decreased DNMT1-induced methylation and loosened the promoter of IGF2 to enhance its expression, subsequently facilitating embryonic growth and liver development.^201^ During zebrafish liver development, the nuclear morphology of hepatocytes was changing. Meanwhile, the chromatin accessibility of development-related genes in the larval liver was enriched. UHRF1 and DNMT1 are required for maintaining appropriate nuclear morphology, and their mutation lead to DNA hypomethylation, loss of lamin B2, and large dysmorphic nuclei in hepatocytes.^234^ Hepatocyte and cholangiocyte are the main cell types that originated from bipotential hepatoblast in liver.^235^ Yang et al. constructed the histone modification and chromatin accessibility profiles during hepatoblast differentiation. They found that the differentiation pathways of hepatoblasts can be determined by chromatin accessibility patterns which were mostly synchronous with H3K27ac on enhancers, and H3K27me3 on promoters. EZH2 and JMJD3, which methylate or demethylate H3K27, respectively, had distinct functions on hepatoblast-to-hepatocyte or hepatoblast-to-cholangiocyte differentiation, while the histone acetyltransferase P300 promoted both progresses.^236^

#### Brain development

In 2021, under the BRAIN Initiative Cell Census Network (BICCN) of National Institutes of Health (NIH), a series of outstanding achievements in brain research have been obtained, providing a spatially resolved cell-type atlas of the motor cortex in different mammals depending on the single-cell transcriptomes, epigennetic modification, and chromatin accessibility.^237–240^ The revelation of neurocyte definition and spatial distribution, transcriptomes and epigenetic markers, as well as the differences among mammals, will construct a solid foundation for the investigation of nervous system evolution, development, and functional execution. In 2023, the Bing Ren group comprehensively analyzed the chromatin accessibility in human brain by single-nucleus ATAC-seq (snATAC-seq). They defined many cell types in brain depending on the single-cell chromatin accessibility atlas. They also identified the specific expressed genes in different cell types and their regulatory networks. What’s more, they predicted the disease-relevant cell types for many neuropschiatric disorders.^241^ Similarly, the accessible regions in the neurons of drosophila brain preferentially drive the expression of genes in neuronal subsets which are distinct in different neuronal sub-types.^242^ Herring et al. constructed the single-cell resolution chromatin accessibility atlas of human brain from gestation to adulthood, which revealed the cell types and chromatin dynamics during the mature of human prefrontal cortex. They also defined some regulatory drivers of neurological and psychiatric diseases.^243^

The nervous system is composed of a variety of cell types. Pavlou et al. indicated that the differentiated astrocytes had distinct expression pattern and chromatin accessibility compared with multipotent neural stem cells (NSCs). They found that the inflammatory condition increased the chromatin accessibility and facilitated the expression of inflammatory response genes. The enriched accessible regions were recognized by Rarg and Dlx1, while the reduced regions were recognized by Tcf21.^244^ LHX2 is a well-confirmed regulator both in early development of hippocampal primordium (Hcp) and neocortical primordium (Ncp).^245^ Suresh et al. demonstrated that the chromatin of Hcp was more accessible than Ncp, and consistent with increased active histone marks, H3K27ac, H3K4me1, and H3K4me3, in the enriched loci. Loss of LHX2 didn’t effect the global chromatin accessibility in Ncp but striking reduced the accessibility in Hcp.^246^ Berg et al. identified that the Hopx-CreER^T2^ line was an embryonic origin of adult dentate neural progenitors. They found that these dentate neural progenitors contained a distinct chromatin accessibility signature compared with the mature dentate gyrus. The genes located in the ATAC-seq peaks are mainly enriched in signal transduction and nervous system development, and the top four TF binding motifs are Zfp354c, Bcl6, Zbtb18, and YY1, all of which have been reported to regulate somatic stem cells.^247^ Cerebellar is one of the main parts of human central nervous system and its development is subtly orchestrated. Zhong et al. established the integrative spatiotemporal development landscape of human fetal cerebellar by systematically using spatial transcriptomics, single-cell transcriptomics, and single-cell chromatin accessibility. They found that not only progenitor cells at different locations displayed differential gene expression and chromatin accessibility profiles, but also differentiated neurons showed distinctive spatial-temporal molecular signatures.^248^ Liu et al. indicated that ARID1A orchestrated the chromatin accessibility and expression of neurogenic and cardiogenic genes in hESCs to facilitate neurogenesis and block cardiogenesis.^249^

#### Lung development

In 2022, He et al. established a single-cell atlas of human fetal lung based on multi-omics analysis, including scATAC-seq, scRNA-seq. They identified lung cells in different differentiation states and mapped human lung development accordingly.^250^ Sox9 is one of the important biomarkers of pluripotent cell in the respiratory buds, which can differentiated into both airway and alveolar epithelium.^251^ Based on the chromatin accessibility and expression pattern variation during respiratory bud development, Khattar et al. revealed that PI3K signaling was essential for the epithelial differentiation of Sox9^+^ progenitors.^252^ Little et al. indicated that NKX2-1 had an opposite impact on the cell fate of lung alveolar type 1 (AT1) and AT2 cells. It interacted with different co-TFs to remodel the chromatin accessibility in there cell types.^253^ FoxF1 is a key factor regulating alveolar capillary development. Guo et al. detected the chromatin accessibility atlas in alveolar capillary dysplasia with misalignment of pulmonary veins (ACDMPV). They confirmed the FoxF1 regulatory network in ACDMPV and provided some potential therapy targets for this disease.^254^

#### Development of other organs or cells

Miao et al. established the chromatin accessibility atlas in mouse kidney, and analyzed the differentiation trajectory of nephron progenitor. They found that FoxL1 was sustainedly expressed during nephron progenitor differentiation, and the expression of Hfn4a and Tfap2b was associated with proximal and distal fates, respectively. Additionally, they indicated that the chromatin accessibility feature can reflect the development mechanism of human kidney, meanwhile the H3K27ac and H3Kme1 modifications may be the main regulators.^255^ Erythropoietin (EPO) is an important peptide hormone regulating erythropoiesis. Riou et al. found that erythropoiesis was enhanced in APC and ARID1A co-deletion mice liver. Mechanically, APC deletion activated β-catenin signaling and ARID1A deletion increased the promoter accessibility of EPO, which synergistically boosted its transcription.^256^ Prepro-B is the first stage of common lymphoid progenitor cells differentiation into B-lineage cells. Recent research indicated that PTEN promoted the B lineage differentiation mainly by suppressing PU.1. PTEN loss blocked the prepro-B to B cell differentiation while promoted it differentiation into T and myeloid lineages depending on chromatin accessibility remolding.^257^ Atoh1 is a master TF to determine the fate specification of cochlear hair cells. Through ATAC-seq analysis, Luo et al. identified two novel enhancers of Atoh1 to regulate its expression in cochlear hair cells.^258^ What’s more, the chromatin accessibility plays an important role in the adaptation of people to the local environment.^259^

It is difficult and restricted to investigate human development in vivo, and the organoid technology can solve part of the problems. Wahle et al. established a human retinal organoid to mimic the retinal development. According to the chromatin accessibility profile, they inferred the gene regulatory network contributing to retinal organoid development.^260^ Kanton et al. established stem cell-derived cerebral organoids of human, chimpanzee, and macaque to reveal the substantial changes of human brain during evolution. They analyzed the cell composition and reconstructed the entire course of cerebral differentiation trajectories in organoids by scRNA-seq. They found that the neuronal development of human was slower than that of the other two primates. The pseudotemporal alignment of differentiation paths indicated that the expression of human-specific genes resolved to distinct cell states are along progenitor-to-neuron lineages in the cortex. Further, using scATAC-seq, they confirmed that the variation of chromatin accessibility contribute to these differences.^261^ Similarly, Trevino et al. developed cortical and subpallial spheroids derived from human iPSCs. By comparing the expression and chromatin accessibility profiles with primary human tissues, both of these spheroids can mimic the early development of human forebrain.^262^ However, the research of Herring et al. revealed that there are few postnatal maturity neurons in the long-term brain organoids,^243^ which indicates that the culture system of organoids requires improvement to better mimic the in vivo situation.

### Chromatin accessibility in tissue regeneration

Most of the somatic cells possess a restricted regenerative capacity, and there are significant distinct in each organ. When injured, the tightly regulated regeneration process will be stimulated. In this part, we will discuss the functions and mechanisms of chromatin accessibility in the regeneration processes in different organs.

#### Heart regeneration

The matured cardiomyocytes are almost nonproliferative, blocking cardiac regeneration after injury. The zebrafish heart has a robust regenerative capacity.^263^ Cao et al. constructed the chromatin accessibility, H3K27ac modification, and expression landscapes of zebrafish heart during regeneration. They identified multiple enhancers with varied accessibility and the corresponding TFs.^264^ Wang et al. indicated that Keratin5 (Krt5) altered genome accessibility at the loci of Pax3a, Acta2, and Bmp4 to promote their expression and zebrafish heart regeneration.^265^ Beisaw et al. also found that the AP-1 binding motifs were enriched most significantly in the gain accessibility regions during zebrafish heart regeneration. Inhibiting AP-1 leaded to defects in cardiomyocyte proliferation and decreased chromatin accessibility at cardiac regeneration genes.^266^ Quaife-Ryan et al. constructed the transcriptome and chromatin landscape of infarcted and noninfarcted neonatal and adult mouse hearts. The chromatin accessibility largely mirrored the transcriptional state. Besides, they confirmed that the neonatal hearts had a higher regenerative capacity than that of adult hearts.^267^ Boogerd et al. revealed that ARID1A suppressed YAP-induced proliferation of cardiomyocytes by remodeling the H3K27ac landscape in mice. Inhibiting ARID1A promoted the proliferation of border zone cardiomyocytes after ischemic injury.^268^

Cardiomyocytes reprogrammed from other cell types is a potential strategy for cardiac regeneration.^269^ Zhang et al. reprogrammed human urine cells into cardiomyocyte-like cells by heterogenous expressing MEF2C, MESP1, Tbx5, and MYOCD, but without GATA4. They found that these TFs, especial MYOCD, remodeled the chromatin accessibility landscape to facilitate the expression of multiple cardiac-specifc genes.^270^

#### Liver regeneration

As the metabolic center, the liver is more likely to be exposed to harmful substances, hence the liver maintains an extraordinary regeneration ability to restore its structure and function effectively after damaged. Regeneration failure will lead to severe liver diseases and complications. (Fig. 7)

During liver regeneration, the chromatin structure is dynamically regulated by many epigenetic events. When injured, the hepatocytes dedifferentiate into liver progenitor-like cells (LPLCs) to participate in liver regeneration. Sox2, Oct-3/4, Klf4, and c-Myc (also called Yamanaka factors or 4F) are the key factor that promote the reprogramming of differentiated cells.^161^ Hishida et al. demonstrated that inducible expression of 4F in hepatocytes specifically promoted partial reprogramming of differentiated hepatocytes into a progenitor state which showed a stronger proliferation ability. They found that 4F can promoted the expression of TOP2A to modify the chromatin accessibility of adult hepatocytes.^163^ Li et al. revealed that ARID1A increased the chromatin accessibility of LPLC-enriched genes and consistently facilitated the binding of YAP to boost the regeneration progress. ARID1A deletion hindered the regeneration of injured liver.^271^ However, an earlier research by Sun et al. found that the expression of ARID1A was reduced in regenerating tissues. Hepatocyte-specific deletion of ARID1A increased the tissue repair of injured liver. Compared with WT mouse, the proliferation ability, tissue damage, fibrosis, and organ function following chemical injuries or surgical resection were improved in ARID1A deficiency ones. Mechanically, the absented ARID1A was replaced by ARID1B in the altered SWI/SNF complex which has distinct function on targeting genes. Lack of ARID1A remolded the histone modification and chromatin structure. These variation block the entrance of the differentiation-related TFs, HNF4a and C/EBPα, as well as the cell cycle and mitosis repressive factor, E2F4, to the their target genes. Reduced H3K4me2 marks in the promoters of HNF4a and C/EBPα-targeting genes, while increased H3k4me2 and H3k27ac marks in that of E2F4-targeting genes were observed, which in line with the transcriptional activity of these genes. They also found that global ARID1A knockout potentiated the healing of injured ear soft tissue.^49^ These contradictory results may be due to their different liver injury molds or other unknown reasons, which confirmed the complex functions of ARID1A from another perspective. Additionally, in the *Fah* null mouse repopulation model, the chromatin structure of hepatocytes are altered significantly during liver regeneration. The chromatin accessibility of proliferation related genes, such as CTCF-targeting genes, were increased while that of hepatocyte differentiation and metabolism-related genes, such as HNF4α-driving genes, were decreased significantly in repopulating hepatocytes.^272^ DINO is a damage-induced lncRNA which amplifies p53 signaling by directly interacting with p53 and increases its stability.^273^ Khanal et al. demonstrated that knockout of nuclear receptor subfamily 2 group E member 3 (NR2E3) decreased the expression of DINO by reducing DINO chromatin accessibility and consequently lowered the p53 protein level. Compared with the WT mouse, the NR2E3 KO mouse showed more severe liver injuries and reduced recovery ability under hepatotoxicity.^274^ MDIG is a mineral dust exposure-induced gene that first identified in alveolar macrophages.^275^ The subsequent study confirmed that it is a JmjC domain containing protein and mediates the demethylation of H3K9me3 to H3K9me1.^276^ Recently, Du et al. demonstrated that liver-specific MDIG-deletion significantly prolonged the recovery process of both partial hepatectomy and carbon tetrachloride (CCl_4_)-treated mouse liver. Mechanically, MDIG promoted the OTX2-induced expression of Myc. The MDIG-induced H3K9me3 demethylation of OTX2 promoter enhanced its chromatin accessibility and subsequently facilitated the entrance of Myc to form a positive regulatory loop.^151^

Intercellular transdifferentiation plays an important role in maintaining organism homeostasis. Fibroblasts, with strong plasticity and proliferation ability, are the ideal doner cell for transdifferentiation. HNF4α and FoxAs play important roles in the development and homeostasis of the liver. Horisawa et al. indicated that FoxAs and HNF4α sequentially and cooperatively bind to the loci of liver-specific gene to induce the mouse embryo fibroblast (MEF) to hepatocyte reprogramming procession. The FoxAs, act as PTFs, increase the chromatin accessibility and recruit HNF4α to its target genes.^156^ Hepatic stellate cells (hPSCs) are intrahepatic resident fibroblasts. Ma et al. indicated that the chromatin accessibility, H3K27ac modification and gene expression pattern were different in human primary hepatocytes and hPSC-derived hepatocytes. The expression of thyroid receptor THRB had no significant differences in these two type cells, but THRB binding motifs were significantly enriched in primary hepatocytes compared with that in hPSC-derived hepatocytes. Thyroid hormone T3 treatment increased the binding of THRB with CYP3A4 proximal enhancer to promote its expression and subsequently facilitated hepatocytes maturation. Even so, they found that engrafting the hPSC-derived hepatocytes into undamaged liver of immunocompromised mice didn’t disrupt the normal histology of the liver.^277^ Other research also indicated that the fibroblast-derived hepatocytes had similar morphology and function with that of hepatocytes, which can apply for the reconstitution of damaged hepatic tissues.^278,279^ These research indicated that the fibroblast-derived hepatocytes are important tissue source for liver repair. Except transdifferentiation into hepatocytes, great many of researches have confirmed that forced expression of cell fate-determining TFs can convert fibroblasts into multiple cell types, such as neuronal cells and cardiomyocytes.^280,281^

Other cell types also can transdifferentiation into hepatocytes. Mukhopadhyay et al. confirmed that adult bone marrow progenitor cells, Lin^-^, participated in liver regeneration of hemophilia A mouse model through engraftment and lineage conversion.^282,283^ In the following work, they found that Lin^−^ cells can be partially reprogrammed to hepatocyte-like cells. The Lin^−^-derived hepatocyte-like cell showed a distinct transcriptional pattern compared with the original Lin^−^ cell. Active histone marks (H3K9Ac and H3K4me3) or repressive marks (H3K9me3 and H3K27me3) were enhanced or reduced, respectively, in the promoters of hepatic TFs, such as C/EBPα/β, HNF1α/3α/3β/4α/6, and GATA4, which were crucial for hepatic development. Further, they confirmed that the upregulated demethylase JMJD3, and down-regulated methyltransferase EZH2, were account for the reduced H3K27me3 modification and reprogramming of bone marrow progenitor cells to hepatocytes.^103^

Besides, biliary is an important part of liver. Multiple researches show that hepatocytes also can transdifferentiate into biliary epithelial cells (BECs). Seirup et al. revealed that the primary hepatocytes cultured in vitro undergo dedifferentiation gradually. They demonstrated that the epigenetic modification and chromatin accessibility of primary hepatocytes changed rapidly when cultured in vitro. Additionally, based on the areas of open chromatin, they identified the TFs involved in the different stages of dedifferentiation, including RXR, Fox, STAT, Fos, Jun, and MAF-related factor families.^284^ Katsuda et al. demonstrated that Sox4 promoted the reprogramming of adult hepatocytes to biliary both in vitro and vivo through altering the histone modifications, including H3K27ac, H3K4me1, and H3K4me3. Sox4 attenuated the activity of hepatocyte enhancers and evicted the hepatic TFs, HNF4a and RXRα, meanwhile opened chromatin in biliary-driving regions. They also found that Sox4 and Sox9 were targets of YAP, the key biliary-reprogramming driving factor.^148^ Merrell et al. indicated that the chromatin accessibility was changing during BECs reprogramming. Re-expression of BEC-specific genes and silence of hepatocyte-specific genes were distinctive characteristics during hepatocyte-to-BEC reprogramming. The expression panel of these genes are in accord with the open state of chromatin. The binding sites of hepatocyte-specific TFs, such as HNF4α, FoxA, and C/EBP, were more accessible in hepatocytes. Conversely, in BECs or reprogrammed cells, accessible sites were enriched for biliary-specific TFs, such as TEAD and HNF1β. Additionally, they revealed that AP-1 and NFκB played an important role in hepatocyte-to-BEC reprogramming.^285^ Besides, Har-Zahav et al. demonstrated that the global DNA methylation was reduced during liver-to-pancreas transdifferentiation, which increases the accessible of the pancreatic TF binding motifs. DNMT1 knockdown improved the efficiency of liver-to-pancreas transdifferentiation by increasing the expression of pancreatic-specific genes.^78^

#### Nervous system regeneration

Many diseases and traumatism may damage the nervous system. The regeneration of axons is essential for the functional recovery of the nervous system from injury. However, the axon growth activity is gradually sealed during neurons mature which is partially correlated with reduced chromatin accessibility of axon pro-regenerative genes.^286^ Some animals, such as zebrafish, possess a robust axon regeneration ability induced by injury. The regeneration progress is strictly regulated by the regeneration-associated signaling.^287^ Dhara et al. indicated that the chromatin accessibility landscape was varied during axon regeneration in zebrafish. The binding motifs of Jun were opened to facilitate the expression of target genes and regeneration progress.^288^ Although the regenerative capacity of the central nervous system (CNS) is restricted, the peripheral nerve has a robust regeneration and repair ability in many mammals. The dorsal root ganglia sensory neurons, containing a regeneration-competent peripheral axonal branch and a regeneration-incompetent central axonal branch, are the ideal material to investigate the mechanism of nerve regeneration.^289^ Palmisano et al. established the dorsal root ganglia injury model at the peripheral or central axonal branch to clarify the mechanism leading to the regeneration ability difference between the central and peripheral nervous system. The comprehensive analysis of RNA-seq, ATAC-seq, and ChIP-seq indicated that the regeneration-associated pathway was activated corresponding with chromatin accessibility, H3K9ac, H3K27ac, and H3K27me3 modification variation in the peripheral axonal injury, but there was no significant change in the central axonal injury.^290^

In addition to the organs mentioned above, chromatin accessibility also regulates the regeneration of other organs, such as lung,^291^ pancreas,^78^ kidney,^292^ and bone.^293^ Differentiation, dedifferentiation, transdifferentiation, and proliferation of specific cells play an important role in the homeostasis and regeneration of organs. As summarized above, chromatin accessibility variation has been shown to participate in these processes. Uncovering the function and mechanism of these progresses are of great significance for the treatment of dysplastic disease, as well as for the rapid and effective recovery or replacement of damaged organs.

### Chromatin accessibility regulates function execution

Each organ has its own unique function, and the expression of many tissue-specific genes plays an important role in the maintenance of their functional homeostasis. Multiple researches have indicated that chromatin accessibility is a master regulator of tissue-specific expressed genes.

#### Liver function

Liver is one of the most vital organs in vertebrate, performing multiple functions, including detoxification, lipid metabolism, glycometabolism, bile secretion, hematopoiesis, immunity homeostasis, and so on.

The function of liver is precisely determined by multiple factors, such as mental state, hormonal readiness, positive and negative feedbacks of metabolic materials, and so on. Many metabolic regulatory signals have been shown to control the expression of downstream genes by regulating chromatin accessibility. The nuclear receptor superfamily, including LXRs, FXRs, and peroxisome proliferator-activated receptors (PPARs), are master regulators of metabolic progresses. Bideyan et al. indicated that knock-out of LXRα/β widely remolded the chromatin accessibility and transcription patterns in mice liver. In addition to regulating the expression of their target genes, loss of LXRα/β can also remold the binding motif accessibility of other TFs, such as reducing the accessibility of CTCF/CTCFL, HNF6/CUX2, HNF1/HNF1β, ATF4/CHOP, nuclear receptor family, and Fox family, while enhancing the accessible of DR1/4 motif and nuclear receptor half-site motif (AGGTCA).^294^ Kain et al. indicated that ligand-activated FXR and LXRα enhanced the expression of target genes by increasing their chromatin accessibility. Mechanically, FoxA2 bound to highly condensed chromatin and interacted with ligand-activated FXR and LXRα to remold the chromatin structure. Additionally, FoxA2 reduced the competing binding of PPARα to ensure the activation of FXR and LXRα signals.^155^

Glucocorticoids (GCs) are useful therapeutic for many diseases by activating their receptor GR. NCoR1 and NCoR2 are two nuclear receptor corepressors. Hauck et al. indicated that liver-specific dual loss of NCoR1 and NCoR2 leaded to hypoglycemia in mice. They found NCoR1 and NCoR2 maintained the accessibility of GR binding motif to regulate glucoregulatory genes.^295^ Grøntved et al. demonstrated that occupied by C/EBPβ maintained the permissive of most GR binding sites, required for the GC-induced response.^296^ In mouse liver, the HNF4α binding motifs and GRE lie adjacent within open chromatin regions. Loss of HNF4α decreases the recruitment of GR to GRE by reducing chromatin accessibility which consequently remodels the expression of GCs-responsive genes.^297^ ANGPTL4 increases the transportation of triglycerides from white adipose tissue to liver, participating in hypertriglyceridemia and hepatic steatosis.^298^ Koliwad et al. indicated that ANGPTL4 was a direct target of GR. Dexamethasone treatment increased the expression of ANGPTL4, coinciding with increased DNase I susceptibility and histone H4 acetylation of GRE in ANGPTL4 promoter.^299^ Besides, earlier research has confirmed that estrogen increase the promoter accessibility of vitellogenin gene B1 in male *xenopus laevis* hepatocytes.^300^

Goldstein et al. confirmed that fasting massively remolded the hepatic chromatin accessibility. Thousands of fasting-induced or -repressed DHSs following 24 h of food deprivation were clarified which were strongly overlapped with enhanced or reduced H3K27ac modification. C/EBPβ, GR, PPARα, and cAMP responsive element binding protein 1 (CREB1) are key TFs to regulate the expression of fasting-induced genes.^301^ Korenfeld et al. also found that fasting promoted the expression of many amino acid catabolism genes, most of which were in line with open chromatin.^302^ Caloric restriction improves metabolic adaptation and reduces the occur of many age-associated diseases.^303^ Fan et al. indicated that short-term caloric restriction remolded the chromatin accessibility profile and HNF4α entrance locus to reprogram fatty acid and bile acid metabolism.^304^ SMARCDs, the SWI/SNF factors, also regulate nutrient and metabolic signaling in liver.^305^

Fetal liver is one of the most important early hematopoietic organs. Farlik et al. constructed the hematopoietic lineage based on DNA methylation maps. They found that hematopoietic cells derived from different tissues, and in different differentiation stages exhibited distinct DNA methylation profiles. Compared the chromatin accessibility dataset which published by another research,^306^ they found that in all cell types, the HSC-specific accessible regions had low DNA methylation level. The reduced DNA methylation of differentiation-related regions, which showed higher accessibility, were only detected in differentiated cells but progenitors. Meanwhile, the differentiated cells derived from different progenitors had different hypomethylation patterns.^307^ Zhu et al. determined the differentiation trajectory from hemogenic endothelial cells to HSC precursors by single-cell RNA-seq and ATAC-seq. They found that Runx1 contains two promoters which have distinct accessibility in different differentiation stages. It regulated the transition efficiency of hemogenic endothelial precursors to hemogenic endothelial cells.^308^ Applying similar method, Anna Maria Ranzoni and colleagues identified the differentiation trajectory from HSCs to multipotent progenitors and confirmed three highly proliferative oligopotent progenitor populations. They also found that the changes of chromatin accessibility preceded transcriptional priming during HSCs differentiation.^309^ Hemogen, also called erythroid differentiation-associated gene (EDAG), plays an important role in promoting proliferation and differentiation of hematopoietic cells through activating GATA1, the critical TF for erythropoiesis. Guo et al. indicated that in fetal liver cells, hemogen enhanced the accessibility of GATA1/LDB1 complex-binding motifs by recruiting coactivator SMARCA4 while expelling corepressor NuRD complex.^46^

The liver microenvironment is involved in regulating immune cell differentiation. The epithelial cells can’t prime circulating naïve CD8^+^ T cells for the hindering of endothelial barrier limiting antigen, but the liver epithelial cell is an exception for its slow blood flow, fenestrations, and basement membrane loss, allowing the direct interaction of CD8^+^ T cells with hepatocytes, which usually leads to T cell unresponsiveness and dysfunction.^310^ Bénéchet et al. indicated that hepatocyte-activated CD8^+^ T cells exhibited distinct chromatin accessibility and transcriptome compared with normally activated CD8^+^ T cells, triggering a defective differentiation program.^311^ Besides, hemolysis releases toxic cell-free hemoglobin and heme, while macrophages phagocytize damaged erythrocytes to reverse the toxic effects.^312^ In mouse and human liver, heme exposed macrophages transformed into erythrophagocytes to suppress inflammatory response. Based on the alterations of chromatin accessibility and transcriptome, they found that heme/NRF2 signaling drove the transformation progress.^313^

#### Brain function

Memory is one of the important functions of the brain. Feierman et al. indicated that the H2B variant H2BE is enriched at the promoter regions of synaptic genes, increasing their chromatin accessibility and expression to regulate long-term memory.^314^ The immune homeostasis in the brain is determined by many immune cells, such as microglia and CD4^+^ T cells. Li et al. indicated that the neuropsychiatric-disorder-associated miRNA miR-137 regulated adult microglial homeostasis and brain function by remodeling chromatin accessibility. MiR-137 competitively bound with Pu.1 to block its binding with chromatin, which consequently inhibited the expression of Jun dimerization protein 2 (Jdp2) to impair phagocytosis and pro-inflammatory response in microglia.^165^ Recently, Elizaldi et al. identified a distinct subset of CCR7^+^ CD4^+^ T cells that was similar with the lymph node central memory cells. The chromatin accessibility features at the Bcl6, CD28, and CCR7 loci were exceeding resembled in both types of cells. Reducing the CCR7^+^ CD4^+^ T cell frequencies in the brain significantly activated microglial and neurodegenerative pathways.^315^ Besides, Chang et al. demonstrated that long light exposure remodeled the chromatin accessibility in the hypothalamus of male quail to regulate their reproductive axis activation.^316^

The chromatin accessibility also regulated the function of heart,^317^ ovary,^318^ lung,^319^ and pancreas.^317^ Currently, investigation focusing on the chromatin accessibility and physiological function execution of different organs is still lacking. However, the chromatin accessibility variation under pathological condition can give us many clues to speculate the truth during the physiological function execution.

### Chromatin accessibility regulates the circadian rhythm

The expression of many genes exhibits circadian rhythmicity. The heterodimer BMAL1::CLOCK is one of the core mammalian circadian clock TFs, driving the first circadian rhythms feedback loop and REV-ERBs, which drives the second feedback loop by inhibiting BMAL1.^320^ Zhu et al. demonstrated that during the circadian cycle, ROR and/or BMAL1 promoted decondensation of global chromatin, facilitating REV-ERB entrance to repress circadian gene expression.^321^ Recently, Qu et al. demonstrated that HNF4α remolded the liver-specific circadian transcriptome. Mechanically, HNF4α facilitated the binding of BMAL1 to its target genes by enhancing their H3K4me1 and H3K27ac modification, as well as chromatin accessibility. Besides, they also found that HNF4α has distinct function on the rhythm regulation in other tissues, which hinted that HNF4α was a novel rhythm regulator.^160^ Takeda et al. indicated that RORγ increased the H3K9ac modification and accessibility of Npas2 promoter, cooperating with REV-ERBα to regulate the circadian expression of Npas2.^322^ Hassan et al. demonstrated that FXR deletion reduced the accessibility of some TF binding motifs, including NF-Y/CBF, Sp1, and Klf, which consequently leaded to dysregulation of the bile acid metabolism and circadian rhythm pathways in hepatocyte.^323^ Besides, air pollution (PM2.5) exposure disrupted the chromatin accessibility and expression profiles of circadian genes in liver and brown adipose tissue through remolding histone acetylation.^324^ There researches demonstrated that circadian rhythms and chromatin accessibility mutually regulated each other, but more mechanisms require to be revealed.

### Chromatin accessibility regulates aging

Aging is a physiological process that regulated by genetic, epigenetic, environmental, and other factors. The transcriptome, histone modification, and chromatin accessibility are changing with aging.

Early research has indicated that the liver chromatin of young rats is more sensitive to different DNA endonucleases than that of old rats, indicating globally decreased chromatin accessibility with aging.^325^ Chen, et al. found that the overall variant of histone H3 occupancy was limiting, however, the decreased H3 occupancy regions were enriched in pro-inflammatory genes.^326^ Ding et al. also indicated that the increased chromatin accessibility was due to the upregulation of inflammation and immune response genes in the senescent liver progenitor-like cells (HepLPCs). Meanwhile, they found that the depletion of FOS like 2 (FOSL2) delayed HepLPCs aging, indicating that FOSL2 may be a key TF which regulated by aging-induced chromatin accessibility.^327^ Bozukova et al. found that the chromatin accessibility of promoter regions was increased in aging murine liver, however the transcriptional output was not increased correspondingly. They found that promoter-proximal pausing of RNA polymerase II was significantly reduced in aging liver.^328^ Additionally, Hu et al. revealed that the expression of histone chaperone, NAP1L2 was in creased in the senescent BMSCs both in vitro and in vivo. Mechanically, NAP1L2 recruited SIRT1 to the promoter of osteogenic genes, such as Sp7, Runx2, and Bglap, reducing their H3K14ac modification, chromatin accessibility, and expression to suppression the differentiation of BMSCs into osteogenic.^134^ Hu et al. indicated that ZKSCAN3 inhibited senescence of human mesenchymal stem cells (hMSCs) by maintaining the stability of heterochromatin. However, it is downregulated in the senescent cells which increased the chromatin accessibility and gene transcription leading to repetition-induced sequences.^329^

### Sex-related chromatin accessibility variation

Multiple researches have indicated that there are many sex-determined differences in chromatin accessibility and gene expression patterns. The distinct pituitary growth hormone (GH) secretion and plasma GH profiles, which are pulsatile in males and persistent in females, are the important reason for sex-based differences of gene expression pattern in liver.^330^ Signal transducer and activator of transcription 5 (STAT5) is the critical TF that responds to the periodic stimulation of GH.^331^ Rampersaud et al. indicated that pulsatile GH regulated the male-biased chromatin accessibility to permit the periodic binding of STAT5. They also found that the binding of Bcl6, CUX2, FoxA1, and FoxA2 to their target locus was sex-dependent.^332^ Besides, the chromatin accessibility, TF binding, and expression of long intergenic noncoding RNAs (lincRNAs) were distinct in males and females, which were regulated by GH.^333^ Additionally, Hao et al. demonstrated that the expression pattern of miRNAs in liver are characterized by sex-biased chromatin accessibility. For example, the expression of miR-1948 (locating in male-biased DHSs) and miR-802 (locating in female-biased DHSs) were higher in males and females, respectively.^334^ These studies indicate that the differences in chromatin accessibility are one of the secrets between males and females.

As mentioned above, chromatin accessibility plays an important role in a variety of physiological processes. However, there still are many aspect required to reveal, such as the variation of chromatin accessibility changes during cognitive formation, emotion changes, environmental perception, and so on. Besides, at present, more and more attention has been paid to the role of lifestyles on our health, hence it’s important to investigate the different lifestyles, such as exercise, routine, diet, and stress induced chromatin accessibility variation. Elucidating their detail functions and mechanisms are of great significance for the maintenance of human health and the treatment of different diseases.

## Chromatin accessibility in pathological processes

**Fig. 8:**
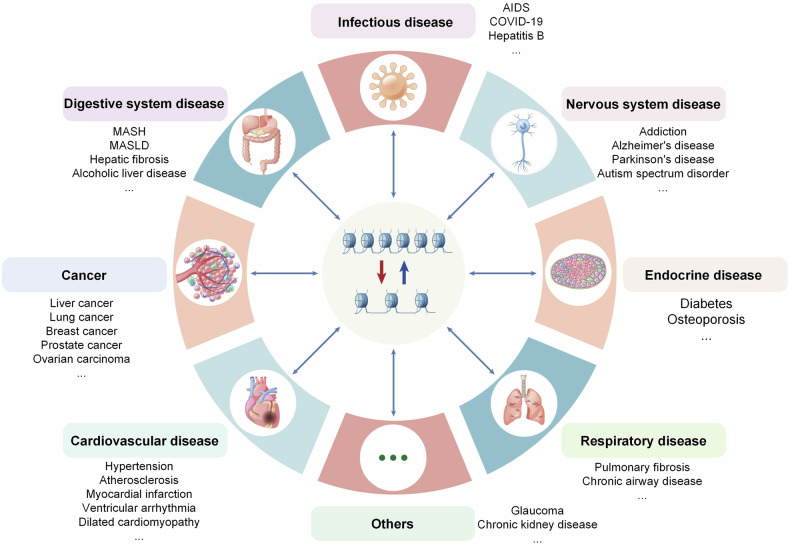
Chromatin accessibility variation is involved in multiple pathological processes. The chromatin accessibility state is dysregulated in multiple diseases. The aberrant chromatin accessibility widely aggravates the initiation and progression of numerous diseases, such as cardiovascular disease, cancer, digestive system disease, nervous system disease, endocrine disease, respiratory disease, and infectious disease. This picture was drawn by Freescience

Epigenetic abnormality is one of the major causes for many diseases, which often leads to anomalous change of chromatin accessibility. The chromatin accessibility is significantly altered in various diseases, indicating that it is an important regulator of disease occurrence and development. In this section, we will discuss the chromatin accessibility variation in different diseases (Fig. 8).

### Chromatin accessibility variation in cardiovascular disease

Cardiovascular diseases are the major cause of mortality across the world. Heart failure is the most common symptom. Dilated cardiomyopathy is one of the major causes of severe heart failure and heart transplantation, which is closely associated with LMNA mutation.^335^ Chromatin accessibility analysis revealed that the TF TEAD1 was critical for cardiomyocyte mature. However, the mutated LMNA gene, coding Q353R-Lamin A/C, hijacked TEAD1 at the nuclear membrane, causing downregualtion of TEAD1 target genes to dysregulate cardiac development.^336^ Shi et al. indicated that the expression of WTAP was decreased both in human and mice heart failure tissues. Specific deletion of WTAP in mice cardiomyocyte (WTAP-CKO) caused dilated cardiomyopathy and even neonatal death. They found that WTAP loss remodeled the chromatin accessibility in the heart. The accessibility of Mef2a/b/c/d binding motifs was decreased while that of TEAD4 was increased in the WTAP-CKO mice. Additionally, WTAP can promote the expression of Mef2c by binding to its promoter directly.^337^ Myocardial infarction is another important inducer of heart failure. Kuppe et al. constructed a spatial multi-omic map of human myocardial infarction. Through snRNA-seq and snATAC-seq analysis, they found that MYH7 and NPPB were upregulated in the pre-stressed and stressed ventricular cardiomyocytes, respectively. Meanwhile, ANKRD1 was increased in both states.^338^ MYH7, NPPB, and ANKRD1 are the confirmed cardiomyocyte-associated stress genes. Hence, their investigation provided an important reference for the research on myocardial infarction. Besides, Kirkland et al. indicated that the loss of Lamin C with age reduced the global chromatin accessibility, downregulated the cytoskeletal regulators and myogenic transcription factors, resulting in age-dependent cardiac decline.^179^ Mitral valve prolapse is a fatal heart disease. Kyryachenko et al. established the chromatin accessibility atlas in human pathogenic and nonpathogenic mitral valves. They identified many mitral valve prolapse risk loci based on the chromatin accessibility variation which provided an indicator for mitral valve prolapse research.^339^ Ventricular arrhythmia is one of the symptoms of long QT syndrome type 7, which is mainly caused by the mutation of KCNJ2 gene.^340^ Recently, Chen et al. repaired the mutant in hiPSCs derived from human patients. Through comparing the chromatin accessibility between mutant or repaired cardiomyocytes during differentiation progress, they found that the accessibility of the TF ZNF528 was significantly increased in the repaired cardiomyocytes. Inhibiting ZNF528 reduced the expression of the pathogenicity associated genes, KCNJ2, CTTN, and ATP1B1.^341^

Vascular dysfunction is another main type of cardiovascular disease. Cheng et al. indicated that specific knockout of ZEB2 in smooth muscle cells accelerated the formation of atherosclerotic plaques in human coronary arteries. ZEB2 insufficiency disrupts both NOTCH and TGFβ signal pathway by remodeling the chromatin accessibility of related genes.^342^ Besides, hypertension widely remodels the chromatin accessibility and expression profiles of vascular smooth muscle and endothelial cells.^167,343^

Cardiovascular diseases always lead to systemic aberrant that involve multiple organs. Uncovering the function and mechanism of chromatin accessibility alteration in cardiovascular diseases will improve the treatment of these diseases.

### Chromatin accessibility variation in cancer

**Fig. 9:**
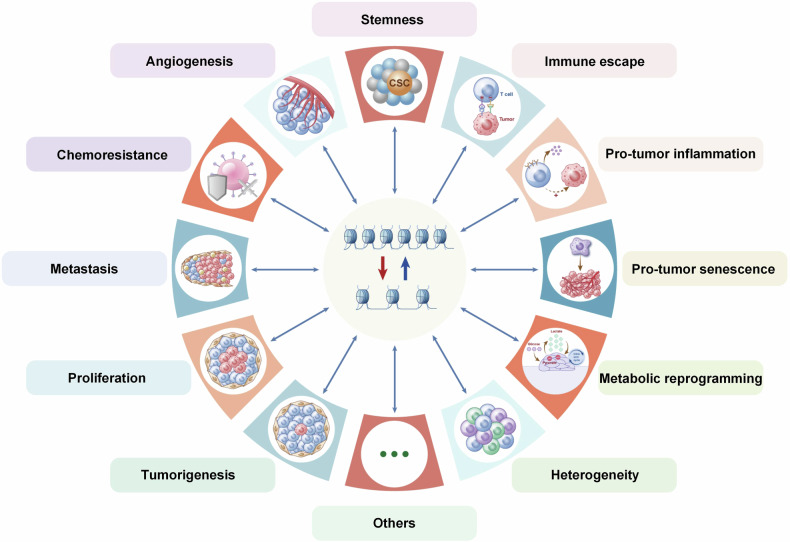
Chromatin accessibility participates in the malignant progression of different cancers. The chromatin accessibility state is dysregulated in multiple cancers which promotes many malignant progression, including tumorigenesis, proliferation, metastasis, chemoresistance, angiogenesis, stemness, immune escape, pro-tumor inflammation, pro-tumor senescence, metabolic reprogramming, heterogeneity, and so on. This picture was drawn by Freescience

Cancer is another leading threaten to human health and life. During the initiation and progression of cancer, the expression profiles of tumor cells are altered significantly which are always in line with the chromatin accessibility state. In 2018, Chang group established the chromatin accessibility landscape of 23 primary human cancers, and screened out lots of cancer risk locus.^344^ In this part, we will discuss the functions and mechanisms of chromatin accessibility alteration in different cancer malignant phenotypes (Fig. 9).

#### Tumorigenesis

Tumorigenesis is a slow and complex process, which is mainly caused by the gradual activation of oncogenic genes and the inactivation of tumor suppressor genes. These progresses are determined by genetic, epigenetic, environmental, and other factors, which always remold the chromatin accessibility in cancer cells.

In liver, MASH is the leading tumorigenesis inducer. Dechassa et al. identified that the accessible chromatin atlas was altered in the MASH-derived HCC. ANXA2 and PDLIM7 were upregulated most significantly which corresponded with enhanced active chromatin marks, H3K4me1 and H3K27ac.^345^ Wu et al. indicated that YAP-TEAD complex promoted the expression of TET1. Consequently TET1 enhanced the DNA demethylation, H3K27ac modification, and chromatin opening of YAP-targeting genes by directly interacting with TEAD. The loss of TET1 reversed the YAP-derived tumorigenesis.^81^ TCPOBOP (1, 4-bis [2-(3, 5-dichloropyridyloxy)] benzene) is a highly specific agonist ligand of the nuclear receptor constitutive androstane receptor (CAR).^346^ TCPOBOP-induced activation of CAR triggers diversity of pathogenic responses in liver. Lodato et al. demonstrated that TCPOBOP induced widely variation in chromatin accessibility and gene expression. The changes in gene expression were in line with the accessibility of chromatin, although the transcription priming of some genes lag behind the chromatin variation.^347^ BAP1 is H2AK119 mono-deubiquitinase. Artegiani et al. indicated that BAP1 loss resulted in aberrant morphology of human ductal liver organoids which showed reduced cell polarity, disrupted epithelium organization, and enhanced motility. Mechanically, BAP1 loss promoted H2AK119ub modification and perturbed the transcriptome pattern of junctional and cytoskeleton components, through regulating the chromatin accessibility of related genes. Restoration of BAP1 activity can rescue its deficiency-induced disorders.^120^

The SWI/SNF complexes can promote or suppress cancer development by binding with different cofactors.^348^ ARID1A is originally hypothesized to be an anti-cancer gene, for it’s the most frequently mutated SWI/SNF component in cancer.^349^ Sun et al. demonstrated that ARID1A has dual function on initiation and development of liver cancer. It promoted tumor initiation in multiple mouse models through enhancing cytochrome P450 induced reactive oxygen species.^48^ In contrast, in the AKT/NRAS-driven hepatocarcinogenesis model, mTORC1 promoted YAP-induced transcriptome by remodeling chromatin accessibility in an ARID1A-dependent manner. Mechanically, mTORC1 directly interacted with ARID1A and enhanced its ubiquitination-mediated degradation. Ectopic expression of ARID1A can antagonize AKT/NRAS-driven hepatocarcinogenesis.^52^ These research indicates the complex function of ARID1A in tumor initiation.

Additionally, chromatin accessibility also regulate the initiation of other tumors, such as lung cancer,^350^ breast cancer,^350^ pancreatic cancer,^351^ colorectal cancer,^352^ and prostatic cancer.^353^ These researches indicate that chromatin remodeling is a significant feature of tumorigenesis and facilitates tumor initiation through various mechanisms.

#### Tumor proliferation

Continuous proliferation is the most significant malignant phenotype of tumor cells. Sun et al. have demonstrated that ARID1A promoted liver tumorigenesis. In the same work, they found that ARID1A inhibited tumor growth and metastasis through decreasing the chromatin accessibility and expression of metastasis-related genes.^48^ MASTL has been report to regulate mitosis in many cancer cells. Liye Cao and colleagues demonstrated that MASTL level was significantly increased in HCC tissues compared with non-tumor liver tissues. IL-6 and TNF-α were able to induce MASTL expression and promoted the proliferation of HCC cells. Mechanistically, upon IL-6 and TNF-α stimulation, the H3K4me3 modification and chromatin accessibility of MASTL promoter were increased remarkably.^145^ NASP is a regulator of cell cycle and proliferation. Kang et al. demonstrated that the expression of NASP was increased in cancer tissues compared with normal tissues and required for the cell proliferation and tumor formation. NASP knockdown decreased H3K9me1 modification and enhanced chromatin accessibility globally which induced the expression of tumor suppressor BACH2 and RunX1T1 to induce replication defect and enhance apoptosis of HCC cells.^147^ The nuclear matrix protein hnRNPU participates in three-dimensional architecture maintenance of hepatocyte genome.^354^ Xu et al. demonstrated that lncRNA RP11-386G11.10 enhanced lipid accumulation by upregulating hnRNPU, promoting the proliferation and metastasis of HCC cells. Additionally, ZBTB7A, the downstream of hnRNPU, promoted RP11-386G11.10 transcription to form a positive feedback loop.^355^ The extracellular matrix (ECM), providing structural and biochemical support to tumor cells, is also closely related with tumor progression. MDIG has been confirmed to promote the regeneration of damaged liver by remolding the H3K9me3 modification and chromatin accessibility of OTX2.^151^ Additionally, researches indicated that the expression of MDIG was usually higher in HCC tissues than normal ones and promoted the growth, metastasis, and drug resistance of HCC cells by promoting the expression of p21 and cell division cycle 6 (CDC6) in a H3K9me3 demethylation-dependent manner.^356,357^

HCC and iCCA are the two main histological subtypes of primary liver cancer derived from hepatocytes or cholangiocytes, respectively. Although they share several common risk factors, there still are distinct characteristics. Recently, Craig et al. compared the chromatin accessibility differences of HCC and iCCA through scATAC-seq. The POU and ETS factors binding motifs were enriched in iCCA while that of nuclear receptors were enriched in HCC.^358^ Besides, Li et al. found that the expression of microfibrillar-associated protein 5 (MFAP5), a ECM glycoprotein, was increasesed in intrahepatic cholangiocarcinoma (iCCA) tissues compared with para-carcinoma tissues. MFAP5 promoted the proliferation of iCCA cells by activating NOTCH1 pathway both in vitro and in vivo. Additionally, they indicated that MFAP5 can remold the chromatin accessibility. The accessible peaks were in line with the upregulated genes, which can be reversed by the NOTCH inhibitor FLI-06.^359^ Recently, Peng et al. indicated that the expression of ΔNp63α was also increased in iCCA tissues and promoted the proliferation, migration, and invasion of iCCA cells. The existence of ΔNp63α increased the accessibility of its binding sites to change the transcriptome. Additionally, ΔNp63α was able to bind with the chromatin structural protein YY1 in iCCA cells.^360^

Additionally, research indicated that loss of H4K20me3 modification correlated with poor prognosis of colorectal cancer (CRC).^361^ Boonsanay et al. demonstrated that the expression of lysine methyltransferase SUV420H2 was decreased in right-sided colon cancer and correlated with poor outcomes. SUV420H2 insufficiency reduced the H4K20me3 modification and increased chromatin accessibility of the Wnt-related genes to promote the growth of cancer organoids.^104^ Beside, chromatin accessibility also regulates the proliferative signaling in lung cancer,^362^ pancreatic cancer,^363^ prostate cancer,^364^ breast cancer,^365^ and so on.

#### Tumor metastasis

Metastasis is the leading cause of cancer-related death. The epigenetics and epitranscriptomics of metastatic tumor cells are altered remarkably. As the most frequently integrated viral gene of HBV, HBx plays an important role in remodeling epigenetic modification during HCC pathogenesis. Zheng et al. indicated that HBx/ETV4/DVL2/β-catenin axis promoted the migration and invasion of HCC cells. Mechanically, HBx promoted the transcription of ETV4 by increasing the H3K27ac modification and chromatin accessibility of super-enhancers upper ETV4 promoter. As a TF, ETV4 promoted the transcription of DVL2.^144^ Wu et al. found that the expression of lncRNA lncMER52A was increased in HCC tissues. The increased expression was due to the enriched H3K4me3 and H3K27ac modification which facilitated YY1 binding to its promoter. Subsequently, sufficient lncMER52A promoted the migration, invasion, and metastasis of HCC cells by suppressing the ubiquitin-mediated degradation of p120-catenin.^366^ Huang et al. indicated that the deficiency of TFAM increased the metastasis of liver cancer. TFAM loss can remold the chromatin accessibility by inducing the polymerization of nuclear actin. The accessible chromatin peaks were enriched in the locus of IL‐6, fibronectin 1 (FN1), TGFβ, integrin β3 (ITGB3), and so on.^181^ Besides, in contrast to promoting tumor initiation, presence of ARID1A inhibited HCC metastasis.^48^

Additionally, the upregulated HDAC8 promoted the brain metastasis of melanoma by enhancing the accessibility of c-Jun binding motifs. It’s interesting that, as a histone deacetylase, HDAC8 increased the H3K27ac modification at these regions by inactivating the acetyltransferase P300. Hence, the deeper mechanisms need to be elucidated.^367^ Through CRISPR-Cas9 screening, Pierce et al. confirmed that LKB1 was a master regulator of chromatin accessibility. Its mutation globally remodeled the chromatin accessibility landscape to drive metastatic phenotype in lung cancer.^368^ What’s more, in breast cancer,^369^ pancreatic cancer,^370^ and colorectal cancer,^371^ the chromatin accessibility variation also is a master indicator and regulator.

#### Tumor cell stemness

In tumor tissue, cancer stem cells (CSCs) are a small group of cells which can promote the treatment resistance, recurrence, growth, and metastasis of many tumors. Many investigations have confirmed that chromatin accessibility variation plays an important role in regulating the stemness of tumor cells.

Wang et al. found that loss of ARID1A increased the proliferation, self-renewal and stemness of hepatocytes. ARID1A deficiency promoted the expression of many CSC-like markers through modifying the chromatin accessibility. Among the verified genes, Jag2 was a key gene that accountable for ARID1A deficiency induced stemness of HCC cells.^50^ HBV infection can hijack the gene expression system of host to ensure viral gene expression and replication, such as inhibiting the expression of RNA helicase DDX5.^372^ The HBV-induced expression of miR106b~25 and miR17~92 directly targeted the 3′UTR of DDX5 to suppress its expression. DDX5 knockdown enhanced viral biosynthesis and CSC-like properties of hepatocytes. Mechanically, DDX5 loss increased the expression and chromatin accessibility of Wnt pathway-associated genes. Inhibition of miR106b~25 and miR17~92 restored DDX5 expression in HBV-infected hepatocytes.^373^ Song et al. reported that in human liver CSCs, circHULC increased the expression of CARM1 by enhancing the interaction of RNA polymerase II with the CARM1 promoter and promoter-enhancer binding loop. The increased CARM1 methylated PKM2 which consequently increased the transcription of SIRT1 to promote the growth of liver CSCs by enhancing autophagy.^374^ The increased promoter accessibility of CBX2 and CEP55 facilitated their expression which consequently triggered stem cell-like phenotype of HCC cells.^375^ Besides, the hypoxia condition and PLD2 overexpression increased the accessibility of stemness genes, promoting the stemness and chemoresistance of ovarian cancer cells.^376^ Hagiwara et al. indicated that MUC1-C, interacting with NRF2 and PBRM1, increased the chromatin accessibility and expression of SLC7A11, G6PD, and PGD to maintain the stemness of prostate cancer cells.^377^

The existence of CSCs is a major challenge in cancer therapy. Now, we can confirm that the chromatin accessibility of CSCs is significantly different with that of common tumor cells, which brings us some promising directions for targeting CSCs.

#### Tumor immune escape

Cancer cells can remold the immune microenvironment to promote cancer progression. The chromatin accessibility is involved in the formation of immunosuppressive microenvironment, resulting in tumor tolerance.

Infiltration of regulatory T cells (Tregs, expressing FoxP3) is the main trigger of cancer immune escape. The CC chemokine receptor 4 (CCR4) is a crucial chemokine receptor that mediates Tregs infiltrating into the tumor microenvironment through binding to its ligands CCL17 or CCL22.^378^ Gao et al. demonstrated that CCR4^+^ Tregs was the predominant Tregs in HBV^+^ HCC, and displayed enhanced immunosuppressive ability. The chromatin accessibility of CCR4^-^ and CCR4^+^ Tregs were distinct, and the expression and promoter accessibility of FoxP3, CCR4, HNF1α, and programmed cell death 1 (PD-1) were increased in CCR4^+^ Tregs. CCR4 N-terminal extracellular recombinant protein (N-CCR4-Fc) or C-021 (CCR4 antagonist) can attenuate CCR4^+^ Tregs-mediated immunosuppression both in vitro and vivo.^379^ Besides, the H3K27ac and H3K4me1 modification landscapes as well as chromatin accessibility were significantly distinct in tumor-infiltrating Treg cells (TITRs) and peripheral blood Treg cells (PBTRs). The expression of genes involved in immune response, cell migration, and leukocyte differentiation were positively concordant with the chromatin accessibility. The accessible motifs that recognized by AP-1 family at the enhancer regions were enriched in TITRs.^380^ Yang et al. revealed that the H3K27ac and H3K4me1 modification landscapes were different in HDAC8 high and low HCC cells. HDAC8 inhibited the infiltration of CD8^+^ T cells by reducing the expression of CCL4, one of the T cell-trafficking chemokines. It reduced the H3K27ac modification of CCL4 enhancer. Knockdown or pharmacological inhibition of HDAC8 increased the infiltration of CD8^+^ T cells and enhanced the therapeutic effect of PD-L1 blockade for HCC.^132^ Zhang et al. found that combined treatment of DNMT and EZH2 inhibitors inhibited proliferation of human HCC cells and upregulated antitumor immune response. The DNMT and EZH2 inhibitors significantly decreased the global promoter DNA methylation and increased the chromatin accessibility, which strongly promoted the expression of immune response and interferon-stimulated genes.^77^ Zhang et al. reported that WD40 repeat-containing protein 6 (WDR6) reprogrammed the tumor immune microenvironment by increasing the expression of TNF-α to promote the growth and metastasis of HCC. It suppressed UVRAG-initiated autophagic degradation of p65 to promote TNF-α expression. Knockdown of WDR6 decreased the chromatin accessibility of the TNF-α locus in Hepa1-6 cells. TNF-α supplementation can revert the WDR6 knockdown reduced infiltration of myeloid-derived suppressor cells.^381^ Chromatin assembly factor 1 (CAF-1), which governs histone H3.1 incorporation into chromatin to facilitate heterochromatin establishment, is crucial for DNA replication during mitosis. Recently, Chan et al. demonstrated that the expression of CAF-1 was increased in HCC. CAF-1 deficiency enhanced antitumor immune response and the treatment effect of immune checkpoint inhibitor. Mechanically, knockout of CAF-1 increased the chromatin accessibility of endogenous retrovirus elements (ERVs), also known as viral mimicry, which activated dsRNA viral sensing and STING pathways in HCC cells.^382^

The innate myeloid cells, including monocytes and macrophages, can be treated to strengthen their immunocompetence.^383^ Wang et al. found that influenza A virus (IAV) infection-treated macrophages gained tissue-specific and long-lasting antitumor immunity and resistant to cancer-induced immunosuppression. They revealed that IAV treatment remodeled the chromatin accessibility atlas of macrophages, among which the accessibility of immune activation genes were increased significantly.^384^

#### Tumor chemoresistance

Acquired resistance to chemotherapy is another challenge in cancer treatment. Tumor cells in the senescent state tend to have higher chemotherapy resistance ability. Zhang et al. indicated that the senescent prostate cancer cells displayed reduced H3K9me2/3 and H3K36me2/3 modification, which were caused by the upregulated demethylases, KDM4. They confirmed that KDM4 promoted the expression of aging-related genes by increasing their promoter accessibility. Inhibiting KDM4 minimized chemoresistance and obtained better prognosis.^115^ Quiescent CSCs are highly resistant to chemotherapy. Yang et al. indicated that the nuclear protein DEK increased the global chromatin accessibility of breast CSCs. Thus, it activated and suppressed the Myc and P53 signaling, respectively, to trigger the quiescence exit of breast CDCs.^385^ Zhang et al. found that many chromatin regions in the anlotinib-resistant lung cancer cells were more accessible than that in the wild-type control, among which, the TFAP2A binding motifs were enriched most significantly. Knockdown TFAP2A sensitized anlotinib-resistant cells to anlotinib through weakening the PDGFR, VEGFR, and TGF-β signaling pathways.^386^ Altered metabolic pattern also contributes to chemoresistance. Ku et al. confirmed that the expression of PRMT1 was increased in pancreatic cancer, remodeling chromatin accessibility and promoting glycolysis. Inhibiting PRMT1 increased the susceptibility to gemcitabine both in vitro and vivo.^387^

#### Tumor heterogeneity

The prominent heterogeneity of cancer cells are also challenging the treatment. Distinct chromatin accessibility status were confirmed in different cancer cell lines, animal models, and patients, which correspond to different malignancy and treatment sensitivity.^388^ B6C3F1 and C57BL/6J mouse have distinct susceptible in phenobarbital-mediated liver tumor promotion.^389,390^ To clarify the mechanism leading to this discrepancy, researchers detected the chromatin accessibility variation of B6C3F1 and C57BL/6J mouse liver after treated by phenobarbital. It showed that these mouse strains maintained differential chromatin accessibility maps in livers after phenobarbital treatment. The B6C3F1-specific DHSs were mainly enriched in Klf4, Wnt/β-catenin, and Src binding motifs, while that enriched in C57BL/6J mainly included Ctnna1, as well as cell cycle, differentiation, and metabolism regulators. The expression pattern of mRNA and protein under phenobarbital treatment was consistent with the chromatin states.^391^ The human hepatoblastoma can divided into well-differentiated pediatric hepatoblastoma Class 1 (C1) and undifferentiated highly proliferative Class 2 (C2) which have favorable or poor prognosis, respectively. The C2 tumors have higher YAP1 expression, proliferation ability, and stem markers than that of C1 tumors.^392^ Smith et al. indicated that conditional expression of YAP1 cooperating with constitutive expression of β-catenin remolded the chromatin structure and drove the formation of hepatoblastoma in vivo, which can mimic the subtypes of human pediatric hepatoblastoma.^393^ In mouse hepatoblastoma model, Rodríguez et al. found that the accessibility of multiple tumor-related cis-regulatory elements had changed in a H3K27ac dependent manner. The YAP1-targeting enhancers were significantly enriched among these open chromatin.^394^ Wang et al. detected the single-cell transcriptomic, proteomic, and epigenetic landscapes of many HCC cell lines. They found that the gene expression patterns and chromatin accessibility were distinct in different cell lines. Based on the chromatin accessibility of the epidermal mesenchymal transformation (EMT) markers, the metastatic potential of HCC cell lines was highly correlated with their EMT scores.^395^ The cancer somatic mutations are tightly associated with DNA replication, and the replication timing are regulated by chromatin structure. The early replicating regions (ERR) are enriched within accessible chromatin and have higher transcription ability, while late replicating regions (LRR) are arranged in the inaccessible portions of the genome.^396^ Yaacov et al. indicated that the mutational profiles of tumors were different between ERR and LRR in multiple cancers including liver cancer. Both replication timing and chromatin accessibility can contribute to mutagenesis.^397^ Chirag Nepal, et al. indicated that many genes in HCC had alternative promoters which possess distinct DNA sequences, DNA methylation, chromatin architecture, H3K4me3, H3K27ac, H3K4me1, and H3K27me3 modifications. The comprehensive function of these variation leaded to the changes in the expression of numerous genes.^398^ Lymphoid neoplasms also are heterogeneous diseases.^399^ Corces et al. identified thirteen human primary blood cell types depending on the chromatin accessibility and transcriptional landscapes. They found significant heterogeneity in these cell types which reflected the hematopoietic differentiation and AML progression.^306^ Rendeiro et al. detected the chromatin accessibility of 88 chronic lymphocytic leukemia (CLL) samples from fifty-five patients which also presented remarkable heterogeneity. In particular, they found that the mutant status of IGHV genes can distinguish the aggressive of CLL. The chromatin accessibility and transcriptional atlas were distinct between the IGHV mutated and unmutanted samples.^400^ Similarly, Wang et al. identified the chromatin accessibility heterogeneity in sixty-one relapsed B-lineage ALL (B-ALL) patients.^401^ In small-cell lung cancer (SCLC), Yang et al. indicated that cancer cells derived from different metastatic model presented distinct chromatin accessibility landscapes which triggered by different transcription factors.^402^ Recently, Yang et al. measured the chromatin accessibility of twenty-three breast cancer cell lines which shown significant heterogeneity and each cell type expressed different key transcription factors to determine their unique chromatin accessibility atlas.^403^

At present, the rising of single-cell omics make us much more easier to understand the heterogeneity of tumor cells.^404^ The various differentiation status of tumor cells is one of the important reasons for tumor heterogeneity. Through applying single-cell multi-omics including ATAC-seq, Roehrig et al. identified a continuum of cell differentiation states in hepatoblastomas (HB).^405^ Desmoplastic small round cell tumor (DSRCT) is definitely mainly triggered by the chimeric transcription factor EWSR1::WT1 but still present heterogeneity. Henon et al. indicated that the accessibility of EWSR1::WT1 binding sites were variational in different DSRCT patients which drove the heterogeneity.^406^ By using similar investigation strategy, many researchers have identified the heterogeneity in other cancers, such as colorectal cancer,^407^ gastric cancer,^408^ and glioblastoma.^409^ Furthermore, the emergence of spatial single-cell omics allows us to reveal the heterogeneity of tumors meanwhile also to define their spatial distribution.^38^ For instance, Mathur et al. constructed a 3D spatial atlas of GBM cells and revealed the spatial feature of genomic, epigenomic, and microenvironmental heterogeneity.^410^ The emergence of various novel technologies make us keep unraveling the mysteries of tumor heterogeneity, however, more efforts are still required to clarify the mechanism and significance, thus proposing novel therapeutic strategies.

In addition to regulating tumor malignant phenotypes mentioned above, chromatin accessibility is also involved in the regulation of tumor metabolic reprogramming,^411^ angiogenesis,^386^ inflammatory response,^412^ and pro-tumor senescence.^413^ In summary, owing to spatial confined, we mainly focused on the functions and mechanisms of chromatin accessibility in HCC, but this does not mean that its role in other cancers can be ignored. Instead, we should devote more efforts to uncover the specific mechanisms of chromatin remodeling in tumorigenesis and development, so as to provide potential targets for more precise and effective cancer therapy.

### Chromatin accessibility variation in liver diseases

Liver diseases are the considerable burden of human health. In the preceding parts, we have summarized the functions and mechanisms of chromatin accessibility in cancer initiation and progression, especially in liver cancer. In this part, we will describe the function and mechanism of chromatin accessibility variant in some common non-neoplastic liver diseases.

#### Lipid deposition in liver

Lipid accumulation is the initiation of many liver diseases. High-fat diet (HFD) can remold the chromatin accessibility in the liver of many vertebrates. Arginine ureahydrolase, arginase 2 (ARG2) is a glucose withdrawal-induced factor, which can partially alleviate the therapeutic metabolic sequelae of caloric restriction. ArcA, a bacterial virulence factor, has higher arginine binding affinity than that of ARG2. Zhang et al. found that both pharmacological activating ARG2 and overexpressing arcA in hepatocyte-induced thermogenesis and insulin sensitivity, attenuated steatosis and inflammation in obesity mice model. Mechanistically, the enhanced arginine catabolism activated systemic autophagic flux and FGF21 secretion. Additionally, scATAC-seq revealed that systemic arginine deprivation altered the chromatin accessibility landscape of hepatocyte and reduced the infiltration of inflammatory macrophagocytes in WT but not in autophagy deficient mice.^414^ The thyroid hormone triiodothyronine (T3) is an important regulator of multiple metabolic progression. The type 2 deiodinase (D2) which expressed in fetal and neonatal mouse liver, activates thyroxine by removing one iodine atom from thyroxine (also called deiodination) to product the active thyroid hormone triiodothyronine (T3).^415^ Fonseca et al. reported that perinatal expression of D2 remolded the gene expression panel and DNA methylation pattern in adult mouse hepatocytes. The mice that hepatocyte-specific knockout of D2 were resistant to HFD-induced obesity, hypertriglyceridemia, and liver steatosis.^86^ Recently, they found that the overall chromatin accessibility was reduced in D2 insufficient mouse which was in line with increased DNA methylation.^416^ Alharthi et al. reported that membrane-bound O-acyltransferase domain containing 7 (MBOAT7) reduced the accessible of inflammatory-related chromatin in COVID-19 and metabolic-associated fatty liver disease. However, MBOAT7 deficiency in metabolic steatohepatitis patients and SARS-CoV-2-induced downregulation of MBOAT7 make it insufficient to inhibit inflammatory responses in these diseases. They also found that MBOAT7 SNP rs8736 could be a disease risk of inflammation-induced liver injure.^417^ Besides, JMJD1C promoted liver lipogenesis both in vitro and vivo under insulin treatment. Mechanically, insulin-induced α-ketoglutarate accumulation and mTOR-mediated T505 phosphorylation of JMJD1C increased its demethylase activity, meanwhile USF-1 facilitated the binding of JMJD1C with the promoters of lipogenic genes. Consequently, JMJD1C enhanced the promoter accessibility of these genes by reducing H3K9me3 modification.^112^

#### Metabolic dysfunction-associate steatotic liver disease (MASLD)

According to the latest consensus, NAFLD and NASH are replaced by MASLD and MASH, respectively. Hepatic steatosis is one of the leading characteristics of many liver diseases. Research has indicated that the initiation and development of fatty-induced hepatopathy are closely accompanied by chromatin accessibility variation. In this part, we will discuss the detailed mechanisms and functions in MASLD.

MASLD is an important subtype of fatty liver diseases, which can develop into MASH, hepatic fibrosis, cirrhosis, and even liver cancer. The mitochondrial signaling is a master epigenetic regulator, dysfunction of which promotes MASLD.^418^ In hepatocytes, mitochondrial stress promoted the expression of AREG, a EGFR ligand. Mechanically, mitochondrial stress increased transcription activity of c-Jun and YAP1 to increase H3K4me1 and H3K27ac modification, as well as accessibility of AREG enhancer region.^146^ Yin et al. found that RPA1 was down-regulated in the livers of fatty liver patients. The loss of RPA1 promoted occurrence of hepatic steatosis, fatty liver, and even spontaneous HCC. Hepatic deficiency of RPA1 suppressed the transcription of FAO-related genes through modulating the chromatin accessibility landscape, and subsequently inhibited FAO.^419^ Bone morphogenetic protein 9 (Bmp9), is a member of the BMP superfamily. It is reported that Bmp9 was down-regulated in HFD fed mice. Ablation of Bmp9 induced lipid accumulation in HCC cells and promoted liver steatosis through reducing the expression of PPARα.^420^ Sun et al. demonstrated that Bmp9 attenuated HFD-induced obesity, hepatic steatosis, and macrophage infiltration by reducing the expression of Cers6, Fabp4, and Cidea, which is involved in glucose and lipid metabolism, as well as Fos, Tlr1, and CCL2, which involved in inflammatory response. Mechanically, Bmp9 treatment reduces the promoter accessibility of Cers6, CCL2, Fabp4, and Fos, meanwhile inhibits TNF and Toll-like receptor signaling pathway.^421^ The aberrant glyoxylate metabolism also promotes the progress of steatosis. Alanine-glyoxylate aminotransferase (Agxt) is a key enzyme in glyoxylate detoxification. Gianmoena et al. indicated that Agxt was downregulated in mouse and human steatotic hepatocytes which was insufficient to detoxificate the mitochondrial glyoxylate. They found that the hypermethylation of Agxt locus reduced its chromatin accessibility in steatotic hepatocytes, and rescue expression of Agxt reduced the production of oxalate in the fatty liver.^422^ Recently, Yang et al. indicated that KDM1A was upregulated in MASLD tissues and promoted the expression of lipid metabolism, glucose metabolism, and inflammation-related genes by increasing their loci accessibility.^113^

Increasing researches demonstrate that Th17 cells promote the pathogenesis of multiple inflammatory disease, such as Crohn’s disease,^423^ cardiovascular disease,^424^ and experimental encephalomyelitis.^425^ Recently, researchers characterized a CXCR3^+^ population of inflammatory hepatic Th17 cells (ihTh17) which were sufficient to exacerbate the pathogenesis of MASLD in mouse. The pathogenic potential of ihTh17 correlated with increased chromatin accessibility, glycolysis, IFNγ and TNFα production. In ihTh17, the open chromatin was enriched in the regulatory regions of metabolic and immune activation-related genes, including CXCR3, STAT1, GAPDH, and PKM2. Intercepting glycolysis by 2-DG or cell-specific deletion of PKM2 reversed ihTh17-induced inflammatory and MASLD severity. Moreover, the ihTh17-like cells are also enriched in human MASLD liver, and positively correlate with MASLD severity. However, the source of ihTh17 cells and the mechanisms of their temporal emergence and maintenance are still unclear.^426^

#### Metabolic dysfunction-associated steatohepatitis (MASH)

Many factors can lead to hepatitis, such as severe hepatic steatosis, excessive drinking, metabolic dysfunction, hepatitis virus infection, autoimmune, and drug damage. According to the latest consensus, the metabolic disturbance-induced steatohepatitis is collectively named as MASH instead of NASH.

MASH is becoming the most important subtype of hepatitis. The variation of chromatin accessibility is one marked feature of MASH. Li et al. found that the protein level of METTL3 was increased in the cytosol but significantly decreased in the nuclei of MASH hepatocytes. They indicated that reduced nuclear METTL3 protein contributed to the MASLD-to-MASH transition in vivo. Specific deletion of METTL3 in liver increased the expression of CD36 and CCL2 which enhanced free fatty acid uptake or inflammation response, respectively. Mechanically, METTL3, interacting with HDAC1/2, removed the H3K9ac and H3K27ac modification of CD36 and CCL2 promoters to suppress their transcription. Hence, insufficient of nuclear METTL3 increased the chromatin accessibility of CD36 and CCL2 promoter. Overexpression of METTL3 or inhibition of CD36 and CCL2 can ameliorate MASH progression.^131^ Pérez-Schindler et al. revealed that the molecular signatures were similar in MASH and hepatocyte lipotoxicity, but they had distinct impact on chromatin accessibility in liver. The global chromatin accessibility of MASH patients was increased slightly, but decreased strongly in hepatocyte lipotoxicity. They indicated that MAF bZIP transcription factor K (MAFK) and transcription factor 4 (TCF4) can contribute to the expression of lipotoxicity-related genes in hepatocyte.^427^ Patatin-like phospholipase domain-containing 3 (PNPLA3), which resides in the membrane of lipid droplets, remodels the lipid droplets after eating through its triglyceride hydrolase activity. The I148M mutant PNPLA3 (PNPLA3 I148M) has less than 20% catalytic activity and higher protein stability, but still accumulated to the surface of lipid droplets to block triglyceride hydrolysis.^428^ Schwartz et al. revealed that momelotinib reduced the expression and promoter accessibility of PNPLA3 in human and mouse hepatocytes, as well as human hPSCs. Momelotinib treatment reduced BMP signaling-induced expression of PNPLA3 and alleviated the PNPLA3 I148M-mediated triglyceride accumulation in MASH model.^429^

Choline-deficient, L-amino acid-defined, high-fat diet (CDAHFD) has been proven to rapidly replicate the full pathologies of MASH, including steatosis, inflammation, and fibrosis of liver. Xiong et al. demonstrated that liver-specific hnRNPU inactivation exacerbated CDAHFD-induced MASH, including enhanced inflammation response and hepatocyte injury. HnRNPU inactivation remolded the chromatin accessibility and transcriptome in hepatocyte, such as the CYP4A10 locus was significantly reduced and Ntrk2 was enhanced in hnRNPU LKO livers. Ntrk2 encodes the TrkB receptor tyrosine kinase. They found that hnRNPU deficiency promoted the expression of a truncated isoform of TrkB, named TrkB-T1. TrkB-T1 can aggravate stress-induced hepatocyte injury. Besides, increased TrkB-T1 expression was observed in liver biopsies of MASH patients. Brain-derived neurotrophic factor (BDNF), the ligand of TrkB can inhibit the expression of TrkB-T1 to relieve liver injury. They speculated that BDNF could trigger endocytosis and degradation of TrkB-T1.^180^ Besides, treating zebrafish larvae with thioacetamide (a hepatotoxin) can be applied to establish steatohepatitis model.^430^ Research indicated that thioacetamide treatment remolded the chromatin accessibility landscape of zebrafish liver cells, especial endothelial cells.^431^

Additionally, liver lacking ARID1A is more susceptible to fatty hepatopathy. Moore et al. indicated that ARID1A deficiency increased lipogenesis and inhibited FAO by disorganizing the expression homeostasis of related genes. Suppressing de novo lipogenesis by SREBP inhibitor delayed ARID1A and PTEN loss-triggered fatty liver. ChIP-seq indicated that the ARID1A binding peaks were significant associated with C/EBPα binding peaks, H3K27ac and H3K4me3 modification in the promoters of most upregulated genes.^432^ Additionally, Qu et al. reported that hepatocyte-specific deletion of ARID1A significantly increased the susceptibility of hepatic steatosis, insulin resistance, and inflammation response in HFD-feeding mice. Mechanically, ARID1A deletion decreased H3K4me3 modification and chromatin accessibility on the promoters of FAO-related genes.^149^ Besides, the SWI/SNF complexes SMARCA2 and SMARCA4 have also been reported to promote the proinflammatory cytokine production in MASH mouse by remolding epigenetic modifications.^433^

In addition to mentioned above, other factors also regulate the chromatin accessibility in MASH liver. Vertical sleeve gastrectomy (VSG) is one of bariatric surgeries which is effectively to improve MASLD and MASH by promoting weight loss. Du et al. demonstrated that VSG reduced the expression of genes in inflammatory pathways and induced the expression of lipid metabolic genes. VSG remolded the chromatin accessibility which was in line with the expression pattern of related genes.^434^ Kupffer cells, the liver resident macrophages, regulate the progression liver diseases, including MASLD and MSAH. Seidman et al. indicated that MASH-induced Kupffer cell death recruited blood monocytes, leading to increased myeloid cells diversity in liver. MASH remolded the chromatin accessibility of Kupffer cells and selectively impaired LXR signaling pathway.^435^

#### Alcoholic liver disease

Excessive alcohol consumption always leads to alcohol-related liver disease, including alcoholic fatty liver, hepatitis, and so on. The alcohol induced epigenetic modification variation is one of the main mechanism. Massey et al. indicated that the hepatic metabolome, especially carbohydrate metabolism of alcoholic hepatitis patients was dysregulated which partially promoted the expression of hexokinase domain containing 1 (HKDC1). The H3K4me1, H3K4me3, and H3K27ac modification of HKDC1 promoter was enriched significantly in alcoholic hepatitis patients.^436^ Increased infection susceptibility is a remarkable characteristic of severe alcohol-related liver disease. Weichselbaum et al. found that the function of monocytes and dendritic cells was impaired in patients with severe alcoholic hepatitis and the pro-inflammatory cytokines were correspondingly reduced, leading to a higher infection and mortality risk. Further, they indicated that compared with healthy control, monocytes derived from patients with severe alcoholic hepatitis displayed a distinct chromatin accessibility landscape which was in line with an immunosuppressive transcriptome pattern.^437^ However, at present, the chromatin accessibility landscapes in alcohol-related liver diseases are still unclear. As alcohol consumption is one of the leading causes of liver diseases, the research about this respect is of great significance.

#### Viral hepatitis

Chronic HBV infection is one of the leading etiologic inducement for HCC. The hereditary material of HBV, called cccDNA, forms microchromosomes in the hepatocyte nuclei. HMGN1 promotes HBV replication and expression of virulence proteins. It increased the accessibility and transcription of cccDNA by competitively combining with H1 and promoting H3 phosphorylation, respectively.^95^ As the most frequently integrated viral gene, HBx plays an important role in HCC pathogenesis which partial through remolding the epigenetic modification.^438^ Zheng et al. indicated that HBx/ETV4/DVL2/β-catenin axis promoted the migration and invasion of HCC cells. Mechanically, HBx promoted the transcription of ETV4 by increasing the H3K27ac modification and chromatin accessibility of super-enhancers upper ETV4 promoter. As a TF, ETV4 promoted the transcription of DVL2.^144^ Additionally, HBV infection reduces the expression of DDX5 to increase the chromatin accessibility and expression of multiple genes involved in Wnt signaling pathway in HCC cells.^373^ Besides, the HBV-induced differentiation of CD8^+^ T cells can be primed by Kupffer cells or hepatocytes, but the latter possesses negligible immune activity. Bénéchet et al. indicated that they have different chromatin landscapes, contributing to the difference in immune activity.^311^ Yates et al. demonstrated that chronic HCV- and HIV-infection exhausted CD8^+^ T cells. Their infection induced an irreversible chromatin accessibility variation in CD8^+^ cells, disrupting TOX and HIF1α signaling.^439^ However, whether HBV infection has a similar effect requires further investigation.

At present, the association between chromatin accessibility and other hepatitis viruses, including HAV, HCV, HDV, and HEV, is rarely reported. Considering the important role of these hepatitis viruses in a variety of liver diseases, it’s of great significance to further investigate the chromatin dynamics regulated by these viruses.

#### Hepatic fibrosis

Uncontrolled hepatitis often progresses to liver fibrosis, even cirrhosis and liver cancer. Research has confirmed that the chromatin accessibility is gradually changing from healthy, steatosis, to fibrotic liver.^440^

The hPSCs only comprise ~10% of the liver, but great numbers of studies have confirmed that their abnormal activation and proliferation are one of the primary triggers for liver fibrosis. Countering with our initial intuitive, Brougham-Cook et al. indicated that the expression of intracellular collagen I and lysyl oxidase (LOX) in activated hPSCs were higher in soft microenvironments (1 kPa, mimicking healthy tissue) than stiff microenvironments (25 kPa, mimicking fibrotic tissue).^441^ Their following work demonstrated that the global chromatin accessibility of hPSCs cultured in soft substrates was higher that in stiff substrates. Among which, the C/EBPβ binding motifs were significantly enriched and the HSD11b1 promoter was more accessible in soft substrate-cultured cells. Correspondingly, knockdown of C/EBPβ or HSD11B1 alleviated fibrogenic phenotype under soft condition.^442^ However, more investigations have indicated that the activation of hPSCs requires a stiff environment.^443–445^ Hence, the detailed ECM condition that regulates the chromatin accessibility in liver requires further research. Liver sinusoidal endothelial cells also play an important role in maintaining the normal function of liver. Winkler et al. found that sinusoidal capillarization was increased significantly in mice that endothelial-specific knockout GATA4, meanwhile these mice showed increased profibrotic angiocrine, hPSC overactivation, perisinusoidal liver fibrosis, and reduced liver regeneration. Further, they revealed that Myc and PDGFB were direct targets of GATA4. Endothelial GATA4 deficiency increased the accessibility of Myc and PDGFB gene loci which subsequently increased their expression. PDGFB is a target of Myc, they also found that GATA4 deficiency increases the binding of Myc to PDGFB promoter.^446^

Transforming growth factor-beta (TGFβ) is a master regulator of fibrosis in many organs. Aseem et al. indicated that TGFβ treatment remolded the landscapes of H3K27ac and H3K9ac modification, and promoted the expression of hPSC-activating genes, such as SERPINE1 and FN1 in cholangiocytes. By comparing the differentially expressed genes enriched by H3K27ac and H3K9ac modification, they found that the H3K9ac enriched genes are more specific. The H3K9ac modification enhanced the chromatin accessibility of TGFβ-stimulating genes. Mechanically, SAMDs, downstream of TGFβ signaling, recruited the acetyltransferase KAT2A to increase the H3K9ac modification in the promoters of target genes. Pharmacologic inhibition of H3K9ac or knockout of KAT2A alleviated biliary fibrosis in vivo.^125^ Besides, Tang et al. demonstrated that the expression of YBX1 was increased in several liver fibrosis models, and aggravated CCl_4_-induced liver fibrosis through activating hPSCs and extracellular matrix deposition. It promoted the expression of multiple fibrosis-related genes by increasing their chromatin accessibility.^447^

#### Other liver diseases

Primary sclerosing cholangitis (PSC) is an immune-mediated liver disease and the ingredient of intrahepatic T cells is aberrant in PSC patients. Poch et al. identified a novel T cell cluster named tissue-resident naive-like CD4^+^ T cells which were expanded in the liver of PSC patients. According to the transcriptome and T cell receptor, this population is similar to circulating naive T cells, but expressed a set of tissue residency genes. Through comparing the accessible chromatin regions of naive CD4^+^ T cells from PSC patients and healthy donors, they revealed that the PSC-derived naive CD4^+^ T cells were predisposed to polarize toward Th17 cells. Several genes that regulate T cell activation and Th17 polarization were identified, such as STAT3, MAF, TLE1, and RUNX1.^448^ In polycystic liver disease, Ji et al. found that the overall chromatin accessibility was increased significantly which activated cyst-associated genes in male and female cholangiocytes. Interestingly, there were significant different manifestations between females and males. The increased chromatin accessibility in male was induced by H3K9me3 elimination and H3K9ac enrichment, and in female is due to H3K27ac enrichment.^449^ In channel catfish, *aeromonas hydrophila* infection increased the serum cortisol levels which regulated the expression of infection-induced genes in liver by affecting chromatin accessibility.^450^ Iron homeostasis is critical for many physiological processes, dysregulation of which leads to many diseases. A recent study indicated that excess iron in liver cells increased the chromatin accessibility of cell cycle genes, most of which are tightly linked to HCC.^451^ Graft-versus-host disease (GVHD), caused by alloreactive T cells infiltration, is the one of the main adverse reactions of organ transplantation.^452^ Inhibitor of DNA binding 3 (ID3) is a DNA-binding inhibitor that regulates T cell fate determination. Wang et al. indicated that ID3 promoted GVHD in the mouse model. ID3 inhibited the expression of PD-1 by reducing its promoter accessibility to facilitate the infiltration of alloreactive T cells. ID3 knockout led to increased liver infiltration of PD-1^+^ T cells. It’s of great meaningful that targeting ID3 reduced the GVHD of CAR-T cells transplantation but don’t compromise the anti-tumor activity.^453^

Liver is one of the most important organs of human being, playing many vital roles, and its lesions often cause serious outcomes. The studies discussed above have partially revealed the functions and mechanisms of chromatin accessibility variation in some liver diseases. However, in numerous other liver diseases, such as bacterial infection, parasitic disease, drug-induced liver damage, autoimmune liver disease, and so on, the chromatin accessibility-related function and mechanism remain to be elucidated.

### Chromatin accessibility variation in nervous system disease

The nervous system disorders are affecting more than one third of the world’s population. These disorders are complex and multiinducible, making it is hard to clarify the specific regions matching disorders. Fortunately, the correlation between the accessibility of specific region and neurological disease is revealing constantly.

Alzheimer’s disease (AD) is a degenerative disease of the central nervous system (CNS), which is the most common form of dementia, mainly occurring in old or pre-old age. Many researches have identified the chromatin accessibility feature in AD patients, screened out many AD risk genes.^192^ The differences in cis-regulatory elements (CREs) are perceived as the main cause of phenotypic differentiation among primate relatives.^454^ Recently, Hu et al. identified many highly conserved rapidly evolving conserved elements (RECEs) in primates which regulated the development of adult human brain. Additionally, they found that these RECEs were generally more accessible in the oligodendrocytes from AD patients compared with control group, indicating that these RECEs may drive brain aging and AD.^455^ Zhang et al. indicated that the postmortem brain of AD patients contained much more double-stranded DNA breaks (DSBs) compared with that in non-demented individuals. These DSBs leaded in unique SNPs, chromatin accessibility, and gene expression profiles in AD brains.^456^ Through analyzing the different open chromatin regions in neurons and non-neurons brains, Bendl et al. found that the binding motifs for USF2 were perturbed most significantly. Downregulation of USF2 disturbed the lysosomal function which was corresponded with the accumulation of abnormal proteins in AD.^192^

Autism spectrum disorder (ASD) is a common neurodevelopmental disorder, and the dysregulation of CHD8 is the most important causal factor.^457^ Shi et al. indicated that CHD8^+/−^ neurons showed a globally increased chromatin accessibility. The genes with varied chromatin accessibility and expression are similar with the genes mutated in probands for ASD, schizophrenia, and intellectual disability. Further, they found that insufficient of CHD8 reduced functional synapses and leaded to reduced spontaneous firing rates in neurons.^458^ NASP is a histone-binding protein occurring in two major forms, sNASP and tNASP which is expressed in somatic cells or testis and embryonic tissues, respectively.^459^ It has reported that NASP regulates chromatin accessibility in HCC cells.^147^ Recently, Zhang et al. identified a nonsense mutant of NASP (tNASP-Q289X) in a Chinese nuclear family with ASD. They found that both tNASP KO and tNASP-Q289X increased the chromatin accessibility and affected the gene expression of neural and immune response signal in the brain.^460^

Additionally, addiction is an obstacle for heroin to treat human disease. FYN, a member of the protein-tyrosine kinase family, regulates the drug-seeking behavior and relapse.^461^ Egervari et al. found that heroin remodeled the chromatin accessibility in striatum. Especially, the accessibility of FYN promoter is increased in the heroin users compared to controls.^462^ Chronic cerebral hypoperfusion (CCH) can activate microglial to promote chronic inflammation-induced neuronal impair.^463^ Zhang et al. indicated that CCH remodeled the chromatin accessibility profile in the cortex, and the PU.1 binding motifs were enriched most significantly in the ATAC peaks. The regulator network of PU.1 may provide potential targets for the early intervention of CCH-induced neuroimmune response.^464^

Besides, other CNS disorder are also involved in chromatin accessibility variation, such as Parkinson’s disease,^465^ stroke,^466^ infection,^315^ and so on. However, the detail roles and mechanisms are required further investigation.

### Chromatin accessibility variation in diabetes

Diabetes is a chronic endocrine disease, characterized by persistent hyperglycemia. The mainly cause of diabetes including insulin secretion defect and cells respond poorly to insulin which leading to type 1 diabetes (T1D) and type 2 diabetes (T2D), respectively. The chromatin accessibility is a master regulator of diabetes.

Autoimmune-induced β cells cytolysis is the main pathogenesis of T1D, and the β cell-specific CD8^+^ T cells maintained stem cell memory phenotype which preserved for long periods, challenging the pancreatic islet transplant.^467^ Abdelsamed et al. confirmed that the β cell-specific CD8^+^ T cells possessed a similar DNA methylation and chromatin accessibility landscapes compared with naive CD8^+^ T cells to maintain a stem-like state.^468^ The haploinsufficiency of BACH2 is main cause of congenital autoimmunity and immunodeficiency.^469^ Robertson et al. screened out many novel T1D risk variants by genome-wide association studies, and by mapping the chromatin accessibility variation, they confirmed that SNP rs72928038 at the promoter of BACH2, leading to reduced promoter accessibility in CD4^+^ T cells.^470^ Similarly, Chiou et al. confirmed that the SNP rs7795896 in the enhancer of CFTR reduced its accessibility and expression in pancreatic ductal cells, indicating a high-risk of T1D.^471^

Currently, there are more studies focused on T2D. Wang et al. indicated that T2D suppressed the insulin production and secretion signaling pathways in pancreatic β cells.^472^ Xue et al. knocked out twenty T2D-associated genes in β cells, and they found that the loss of many genes can remodel the chromatin accessibility and expression profiles significantly. Among which, HNF4α was a key TF for the maintenance of β cells physiological functions. Besides, CP, PCSK1N, RNASE1, and GSTA2 regulated insulin production. TAGLN3 and DHRS2 regulated the lipotoxicity sensitivity of β cells.^473^ Qiao et al. indicated that knockout STING suppressed Pax6 expression and nuclear localization, while the accessibility of Pax6 targets was correspondingly reduced, leading to disturbed glucose-stimulated insulin secretion.^474^ Reduced IGF2BP2 expression and accessibility of its binding sites may contribute to T2D.^475^ Glucocorticoid also increases the chromatin accessibility at glucocorticoid receptor binding sites to regulate the expression of T2D-associated genes.^476^ Besides, Qadir et al. indicated that the sex-based difference in gene accessibility and expression were predominantly enriched in sex chromosomes of cultured islets derived from nondiabetic donors, meanwhile in islets from T2D, there was much more sex-based difference in autosomes, and the females displayed more variation compared with male donors. The differentially expressed genes in males and females were enriched in insulin secretion pathways, oxidative phosphorylation and electron transport chain pathways, respectively, which corresponded with the sex-based pathogenesis of T2D.^477^ Additionally, Wang et al. indicated that HFD-feeding remodeled the chromatin accessibility and transcriptome through suppressing CTCF expression, which was corresponded with decreased CTCF expression in β cells from obese and diabetic mice and humans. Further, they confirmed that both early dietary intervention and restoration of CTCF expression reversed β cell dysfunction.^478^ Besides, Wei. et al. indicated that Vitamin D remodeled chromatin accessibility landscape by orchestrating the interaction of BRD7 and BRD9 binding with BAF chromatin remodeling complexes to protect β cells.^479^ Through genome-wide accessibility studies, many T2D-associated variation loci have been screened out in human,^480^ but their role in T2D is required investigation.

Diabetes usually leads to multiple organ abnormalities in patients. Bansal et al. indicated that T2D dysregulated the fibrotic and transport-associated genes in kidney proximal tubule epithelial cells by remodeling their DNA methylation and chromatin accessibility. The most disturbed TF binding sites included HNF4α, SMAD3, and CTCF.^481^ Cavalli et al. detected the difference of chromatin accessibility in normal and T2D livers. Based on the differential accessible regions, they identified a novel enhancer of acyl-CoA thioesterase 1 (ACOT1) gene, which catalyzed the hydrolysis of acyl-CoA to free fatty acid and coenzyme A (CoA). The increased expression of ACOT1 in T2D liver was in line with the more accessible chromatin structure. Meanwhile, multi-omics study indicated that the ACOT1 level was an indicator of T2D.^482^

### Chromatin accessibility variation in other diseases

In addition to mentioned above, the chromatin accessibility variation is also involved in many other diseases. Such as, in T-cell acute lymphoblastic leukemia (T-ALL), the chromatin is more accessible than that in normal human immature T cells.^483^ In idiopathic pulmonary fibrosis myofibroblast, the increased accessibility and expression of TWIST1 promotes fibrosis progress.^484^ Raphael et al. demonstrated that the accessibility of IL-33 promoter was increased in airway cells of chronic obstructive pulmonary disease patients, facilitating IL-33 expression and aggravating the disease.^485^ Besides, chromatin accessibility variation is also involved in the progression of chronic kidney disease,^486^ AIDS,^487^ COVID-19,^488^ osteoporosis,^489^ and so on.

It’s difficult to list all the diseases that occur in our organs and systems, but based on the available research, we can confirm that the initiation and development of numerous diseases are accompanied by chromatin accessibility variation, which are both disease-induced outcomes and disease triggers. Therefore, chromatin accessibility is an ideal indicator of morbidity, target for treatment, and biomarker for prognosis. What’s more, research strategies targeting single organ or single disease are already abundant, however, most diseases are often caused by systemic abnormalities and many diseases also cause systemic lesions. At present, the single-approach treatment often causes severe side effects which seriously reduce the curative effect of disease and the life quality of patients. Therefore, it’s of great significance to ensure the life quality of patients while ensuring the treatment effect of disease by every means. In recent years, the importance of integrative medicine in maintaining human health and disease treatment has obtained more and more attention. Hence, comprehensive research on systemic variation of chromatin accessibility in various disease processes may help to reveal the pathogenesis and development mechanisms of these diseases as well as propose better treatment strategies. Besides, the traditional Chinese medicine (TCM) has an innate advantage in disease treatment through a systemic manner, therefore, it is of great significance to investigate the alteration of chromatin accessibility during the treatment of diseases by TCM. Comprehensively revealing the function and mechanism of chromatin remodeling in different diseases allows us to treat these diseases more timely and effectively, however, there still are many challenges to be solved.

## Targeting chromatin accessibility for disease therapy

As we known, the chromatin accessibility varies significantly during various pathological process, and given its powerful function in the initiation and progression of different diseases, the chromatin remodellers are potential targets for disease treatment. In this section, we will discuss the disease treatment strategies based on targeting different chromatin regulators (Table 1).

**Table 1:** The inhibitors of chromatin accessibility remodellers

Target	Inhibitor	Mechanism	Function	Ref.
SMARCA2/4	ADAADi	Inhibiting the ATPase activity	Altering the epigenome and suppressing neuroblastoma, cervical carcinoma, and prostate cancer.	^490–492^
	BRM014		Varying the chromatin accessibility	^493^
	FHD-286		Inhibiting AML stem cells	^494^
SMARCA2/4	PFI-3	Targeting the bromodomains	Sensitizing cancer cells to chemotherapy	^495,496^
PBRM1	PB16		Unclear	^497^
	GNE-235		Unclear	^498^
BRD7/9	LP99		Evicting BRD7 and BRD9 from acetylated histones	^502^
BRD9	BI-7271		Inhibiting AML in xenograft model	^503^
	BI-7273			
	BI-9564			
	I-BRD9		Suppressing AML	^504^
BRD7	1-78		Inhibiting prostate cancer	^505^
	2-77			
SMARCA4	JQ-dS-4	PROTAC-dependent degradation	Suppressing glioma	^506^
SMARCA2/4, PBRM1	ACBI1		Causing apoptosis of cancer cells	^507^
	AU-15330		Inhibiting prostate cancer in xenograft models and sensitizing to AR antagonist treatment	^508^
SMARCA2	A947		Suppressing SMARCA4 mutant solid tumors	^509^
	PRT3789			^510^
BRD9	GSK39		Inhibiting cancer and immunology-related genes in AML	^511^
	dBRD9		Inhibiting the growth of multiple myeloma cell both in vitro and in vivo	^512^
	C6		Inhibiting the progression of AML	^513^
	CFT8634		Suppressing the growth of synovial sarcoma both in vitro and in vivo	^514^
	FHD-609		Inhibiting the growth and promoting apoptosis of AML cells	^515^
BRD7/9	VZ-185		Unclear	^516^
SMARCA5, CHD4	ED2-AD101	Binding to the HELICc-DExx domain	Sensitizing cancer cells to chemotherapy and inhibiting cancer progression	^517^
MBD2	Mitoxantrone	Inhibiting the interaction of MBD2 with methylated DNA	Unclear	^518^
	Idarubicin			
	NF449			
	Aurintricarboxylic acid			
HDACs	TSA	Competitively binding the catalytic site of HDACs	Increasing chromatin accessibility and CRISPR/Cas9 editing efficiency	^519^
	SAHA		Elevating H3K9 and H4K5 acetylation and increased chromatin accessibility	^520^
HDAC1/2	Cmpd60	Slow-binding, benzamide-based	Alleviate aging	^521^
	MGCD0103	Inhibiting HDAC dependent on benzamide group	Altering the epigenome, inducing apoptosis, and suppressing cancer progression	^522^
	FK228	Suppressing histone deacetylase activity	Enhancing the transcriptional activity of SV40 promoter	^523^
HDAC1	CI-994	Inhibiting HDAC dependent on benzamide group	Altering the epigenome, inducing apoptosis, and suppressing cancer progression	^524^
BPTF	BI-2536	Targeting the bromodomains	Unclear	^526^
	Bromosporine		Inhibiting the proliferation of leukemic cell	^527^
	DCB29		Unclear	^528^
	C620-0696		Inducing apoptosis and cell cycle blockage of NSCLC cells	^529^
RUVBL1/2	CB-6644	Inhibiting the ATPase activity	Suppressing cancer progression	^530^
	Sorafenib			^531^
DNMTs	Azacytidine	Nucleoside analogs which irreversibly binding with DNMTs	Suppressing cancer progression	^532^
	Decitabine			
DNMT3B	Nanaomycin A	Binding with the catalytic site of DNMT3B	Sensitizing HCC cells to sorafenib	^535^
DNMTs	SGI-110	Nucleoside analogs	Inhibiting prostate cancer	^536^
EZH2	GSK126	Competitive with S-adenosyl-methionine	Suppressing HCC	^77^
α-KG-Dependent histone and DNA demethylases	2-HG	Competitive with α-KG	Altering histone methylation in human glioma cell line and tumor samples	^540^
	Fumarate		Altering genome-wide methylation of histone and DNA	^541^
	Succinate			
TET1	Bobcat339 hydrochloride	Cytosine-based inhibitor	Reversing the MAGI2-AS3 induced demethylation of MAGI2 promoter	^545^
P300	GNE-781	Binding with the bromodomain	Reducing the chromatin accessibility of super-enhancers in multiple myeloma	^546^
	GNE-049		Reducing the H3K27ac modification on integrated HIV DNA	^547^
	CBP30		Decreasing the H3K27ac modification and chromatin accessibility of somatic-specific genes	^548^
	I-CBP112			
	A-485	Inhibiting the acetyltransferase domain	Remodeling the enhancer landscape in leukemia stem cells	^549^
HATs	Anacardic acid	Blocking the histone acetyltranferase activity	Reducing the expression of IL-6 in macrophages to alleviate paraquat-induced pulmonary fibrosis	^550^
MOF	MG149	Targeting the Acetyl-CoA binding site	Reducing the expression of NOX1/NOX4 in I/R injury mice	^551^
TIP60			Decreasing the total Khib modification level and suppressing pancreatic cancer	^552^
HDAC8	PCI-34051	Inhibiting the enzymatic activity	Suppressing HCC both in vitro and in vivo	^132^
DOT1L	EPZ-5676	Occupying the S-adenosyl methionine binding pocket and inducing conformational change	Reducing the H3K79me2/3 modification in leukemia cells globally and the accessibility of Sox2-binding enhancers to impair neuronal differentiation	^106,107^
JMJD3	GSK-J4	Binding with the catalytic pocket	Remodeling chromatin accessibility and promoting ferroptosis of HCC cells	^111^
PRMT1	Furamidine	Targeting the enzyme active domain	Reversing the chemoresistance of pancreatic cancer	^387^
AR	Enzalutamide	Inhibiting nuclear translocation	Suppressing prostate cancer	^554^
ONECUT2	CSRM-617	Binding with the HOX domain	Reshaping chromatin accessibility and inhibiting prostate cancer	^555^
Myc	MYCi975	Binding with bHLHZip domain	Promoting degradation of Myc, reducing H3K27ac modification and chromatin accessibility	^556,557^
CDK9	AZD4573	Inhibiting Kinase activity	Remodeling the accessibility of promoters and enhancers, and inhibiting lymphoma cells	^558^
TOPs	Acriflavine	Intercalating into DNA and binding to TOPI/II	Increasing the locus accessibility lncRNAs in endothelial cells	^559^
E2F	HLM006474	Blocking the DNA-binding activity	Remodeling chromatin accessibility and inhibiting glioblastoma tumorigenicity	^560^
STAT3	S3I-201			
KLF5	Am80	Impairing the formation of transactivation complex	Inhibiting skeletal muscle atrophy	^561^
NOTCH2	Gliotoxin	Blocking the DNA-binding activity	Remodeling chromatin accessibility and inhibiting chronic lymphocytic leukemia	^562^

This table only shows the functions of inhibitors in the corresponding references, but the function of most inhibitors is not limited to this

### Therapeutic opportunity by targeting nucleosome remodeller

Nucleosome remodeller can wildly regulate the nucleosome distribution and chromatin accessibility, participating in many pathological processes. Thus, targeting these nucleosome remodellers is an opportunity to treat some diseases.

#### Targeting SWI/SNF complexes

There are many inhibitors have been found and characterized to target different subunits or domains of SWI/SNF complexes. Hydrolyzing ATP as energy is the common characteristic of different nucleosome remodellers. Hence, targeting ATPase activity is the primary strategy to block their function for disease treatment. Researches indicated that insufficient SMARCA2 and/or SMARCA4, the ATPase activity subunits of SWI/SNF complexes, sensitized the cancer cells to chemotherapy or radiotherapy. Active DNA-dependent ATPase A Domain inhibitor (**ADAADi**) is the first identified inhibitor directly targeting SWI/SNF catalytic activity.^490^ It has been reported to suppress the progression of many cancers.^491,492^ **BRM014**, an allosteric ATPase inhibitor of SMARCA2/4 varying the chromatin accessibility very rapidly.^493^ **FHD-286**, another allosteric inhibitor of SMARCA2/4, has been showed to inhibit AML.^494^

Up to date, at least five SWI/SNF subunits bear bromodomains, including Family IV and VIII. The bromodomains are important reader of epigenetic code on chromatin, indicating that targeting the bromodomains can effectively inhibit SWI/SNF complex activity. SMARCA2/4 and PBRM1 bear Family VIII bromodomains. **PFI-3** is the first identified inhibitor selectively binding to the bromodomains of SMARCA2/4 and PBRM1. Although applying **PFI-3** alone has limited effect on some cancer cells, it significantly sensitized of these cancer cells to chemotherapy.^495,496^ **PB16** and **GNE-235** are two promising PBRM1 selective inhibitors, but their biological function remains to be elucidated.^497,498^ BRD7 and BRD9, assembled into different SWI/SNF complexes, contain Family IV bromodomains.^499^ BRD9 or BRD7 is considered as oncogene or tumor suppressive, respectively,^500,501^ thus more studies are focused on targeting BRD9. **LP99** is the first identified drug binding to the bromodomains of BRD7 and BRD9 to evict both proteins from acetylated histones.^502^ The BRD9 selective bromodomains inhibitors, such as **BI-7271,** **BI-7273,** **BI-9564,**^503^ and **I-BRD9,**^504^ significantly inhibit the progression of many cancers. Recently, compounds **1-78** and **2-77** were identified as the first BRD7-selective inhibitors which can suppress the proliferation of prostate cancer cells.^505^

PROTAC is an effective method for specific protein degradation in vivo. Panditharatna et al. constructed an ATPase degrader by linking a SMARCA4 ATPase inhibitor to a phthalimide, called **JQ-dS-4**, facilitating ubiquitin E3 ligase CRBN-mediated degradation, which suppressed the progression of glioma.^506^ **ACBI1** and **AU-15330** are two PROTACs that link the Family VIII bromodomains with E3 ligase VHL to promote the degradation of SMARCA2/4, and PBRM1.^507,508^ **A947** and **PRT3789** selectively degrade SMARCA2 to suppress SMARCA4 mutant solid tumors.^509,510^ **GSK39,**^511^ **dBRD9,**^512^ **C6,**^513^ **CFT8634,**^514^ and **FHD-609**^515^ are CRBN-based PROTACs for BRD9, all of which showed anti-cancer activity. Besides, **VZ-185**, a VHL-based PROTAC, degrades both BRD7 and BRD9.^516^

#### Targeting NuRD complex

The chromodomain helicase DNA-binding proteins (CHDs), containing ATPase activity, are the core subunits of NuRD complexes. Oyama et al. indicated that the SMARCA5/CHD4 dual inhibitor, **ED2-AD101**, sensitized ovarian cancer cells to cisplatin by reducing the expression of multi-drug resistance 1 (MDR1).^517^ MBD2 is a methylated DNA-binding protein within the NuRD complex. Wyhs et al. screened out four inhibitors, **mitoxantrone,** **idarubicin,** **NF449**, and **aurintricarboxylic acid**, to inhibit the binding of MBD2 with methylated DNA.^518^ HDAC1 and HDAC2 are the important subunits of different NuRD complexes. **Trichostatin A (TSA**) and **SAHA** (also called as **Vorinostat**), two broad spectrum HDAC inhibitors, are applied in many clinical trials. Both **TSA** and **SAHA** have been reported to remodel the chromatin accessibility.^519,520^ Up to date, many HDAC1/2 selective inhibitors have been found, such as **Cmpd60,**^521^ **mocetinostat (MGCD0103),**^522^ **romidepsin (FK228),**^523^ **tacedinaline (CI-994),** **santacruzamate A (CAY10683),**^524^ and so on, which have the potential to treat cancer and hypertension, as well as alleviate aging.

#### Targeting ISW1 complex

SNF2H (also called SMARCA5) is one of the core subunits of ISW1 complex, containing ATPase activity. Kishtagari et al. identified a SMARCA5/CHD4 dual inhibitor, **ED2-AD101**, suppressed the proliferation of AML cells and promoted the epithelial differentiation of many solid tumor cells.^525^ BPTF is the largest subunit of ISW1 complex, containing bromodomains. **BI-2536** and **bromosporine** are the first two discovered inhibitors of BPTF, but their specificity are limited.^526,527^ Zhang et al. screened out a selective BPTF bromodomain inhibitor, called **DCB29**, but the biological function is unknown.^528^ Xu et al. identified another BPTF bromodomain inhibitor, named **C620-0696**, which suppressed the progression of NSCLC mainly by inhibiting c-Myc transcription.^529^

#### Targeting INO80 complex

RUVBL1 and RUVBL2, containing ATPase activity, are parts of INO80 and SWR1 complexes. Assimon et al. identified an allosteric inhibitor of the ATPase domain of RUVBL1/2, named **CB-6644**, which can suppress the progression of many cancers.^530^ Besides, Nano et al. indicated that **sorafenib** inhibited the ATPase activity of RUVBL1/2 complex by directly interacting with RUVBL2.^531^

Currently, there are many chromatin remodeller inhibitors, but satisfactory results are rarely achieved in clinical application. What’s more, the functions of most inhibitors on chromatin accessibility, in particular at specific sites, are still require further investigation.

### Therapeutic opportunity by targeting DNA methylation modifier

The methylation of DNA is dynamically regulated by DNA methyltransferases and demethylases which have been identified as inhibition targets for various diseases. DNA methyltransferase enzymes (DNMTs) catalyzed the CpG islands methylation. **5-azacytidine (azacytidine)** and **5-aza-2’-deoxycytidine (decitabine)** are two cytidine analogs that specifically inhibit DNA methyltransferases.^532^ Bowler et al. indicated that **azacitidine** promoted the degradation of DNMTs, in particular DNMT1.^533^ Kapuganti et al. indicated that the promoter of CLU was hypomethylated, leading to reduced expression of CLU in the blood and lens capsules of pseudoexfoliation patients. Treating human lens epithelial cells with **decitabine** promoted CLU expression by facilitating its promoter accessible to the TF Sp1.^534^ **Nanaomycin A** can sensitize HCC cells to sorafenib by inhibiting DNMT3B.^535^ Rodems et al. indicated that DNMT inhibitors **decitabine** or **guadecitabine (SGI-110)** enhanced the antitumor immunity by increasing the accessibility and expression of HLA-I in prostate cancer cells, while combination with the HDAC inhibitor **LBH-589 (LBH)** getting a better effect.^536^ Additionally, combined treating by DNMT inhibitor **decitabine** and HDAC inhibitor **SAHA** increased the chromatin accessibility and sensitized solid tumor cells to cisplatin, doxorubicin, and irradiation.^537^ Combined applying **decitabine** and EZH2 inhibitor **GSK126**, suppressed the proliferation and upregulated anti-tumor immune response in human HCC cells.^77^ Besides, many natural bioactive compounds have been reported to inhibit DNMTs in many cancers.^538^

The removement of 5mC modification dependents on the ten-eleven translocation protein family (TETs) and thymine DNA glycosylase (TDG).^539^ Researches have indicated that the accumulation of oncometabolite, **fumarate,** **succinate**, and **2-hydroxyglutarate (2-HG)** induced by hydratase (FH), succinate dehydrogenase (SDH), and isocitrate dehydrogenase 1/2 (IDH1/2) mutations, respectively, significantly inhibited both α-ketoglutarate (α-KG)-dependent histone and DNA demethylases,^540,541^ which linking metabolism and epigenetics.^542^ Verdura et al. indicated that **decarboxymethyl oleuropein aglycone (DOA)**, a naturally phenolic compound in olive oil, can inhibit the mutant IDH1, IDH1-R132H, to reverse hypermethylation of DNA and H3K9 through restoring the 2-HG-suppressed demethylases.^543^ Multiple researches have indicated that targeting IDH-mutant to restore DNA and histone demethylation is a promising anti-cancer strategy.^544^ Xu et al. indicated that MAGI2-AS3 suppressed the proliferation and migration of breast cancer cells by increasing the demethylation of MAGI2 promoter and facilitating its expression. But, the TET1 inhibitor, **bobcat339 hydrochloride**, reversed the inhibitory effect of MAGI2-AS3.^545^

### Therapeutic opportunity by targeting histone modifier

The epigenetic modifications on histones and their functions are complex, which orchestrated by multiple modifiers. Lots of histone modification-targeting molecules have been characterized and even applied in clinic. Some of which have been reported to remodel the chromatin accessibility.

CBP/P300 is the best-studied acyltransferase, mediating many types of acylation on histones and non-histone proteins. Recently, Welsh et al. indicated that the P300 inhibitor **GNE-781** sensitized multiple myeloma (MM) to immunomodulatory imide drugs (IMiD) through blocking the super-enhancers accessibility and suppressing the expression of Myc, IRF4 and POU2AF1.^546^ Lindqvist et al. indicated that inhibiting P300 by **GNE-049** reversed HIV latency by reducing the H3K27ac modification on integrated viral DNA.^547^ Ebrahimi et al. indicated that the CBP/P300 bromodomain inhibitors, **CBP30** and **I-CBP112**, facilitated cellular reprogramming by decreasing the H3K27ac modification, chromatin accessibility, and expression of somatic-specific genes.^548^ **A-485** inhibits the acetyltransferase activity of P300 to targeting enhancer accessibility in leukemia stem cells.^549^ Besides, Hu et al. revealed that the histone acetyltransferase (HAT) inhibitor, **anacardic acid**, alleviated paraquat-induced pulmonary fibrosis by reducing the expression of IL-6 in macrophages.^550^ **MG149** inhibits MOF to attenuate MKL1-induced cardiac ischemia-reperfusion injury in mice.^551^ **MG149** also decreases the total lysine 2-hydroxyisobutyrylation (Khib) modification by inhibiting TIP60 in pancreatic cancer.^552^

Currently, there are more researches on histone deacetylase inhibitors. Through high-throughput small molecule screening, Pattenden et al. identified a series of HDAC inhibitors, such as **SAHA,** **TSA,** **panobinostat (LBH-589),** **givinostat (ITF2357),** **abexinostat (PCI-24781),** **tubastatin A** (HDAC6 selective), **PCI-34051** (HDAC8 selective), and so on, which can remodel the chromatin accessibility.^553^ Yang et al. indicated that the HDAC8 inhibitor, **PCI-34051**, increased the infiltration of CD8^+^ T cells and enhanced the therapeutic effect of PD-L1 blockade in HCC mice model.^132^ Similarly, the HDAC6 inhibitor, **tubastatin A**, also inhibits the expression of PD-L1 in melanoma cells. Besides, Tiwari et al. indicated that **epigallocatechin gallate (EGCG)** increased the chromatin accessibility by inhibiting HDAC which alleviated radiation-induced hemopoietic system injury in mice.

Targeting histone methylation has also obtained many achievement. Treating with DOT1L inhibitor, **EPZ-5676**, reduced H3K79me2/3 modification globally in leukemia cells.^106^ **EPZ-5676** also reduced the H3K79me2 modification and accessibility of Sox2-binding enhancers to impair neuronal differentiation.^107^ Combined utilization of donafenib and the JMJD3 inhibitor, **GSK-J4**, suppressed the proliferation of liver cancer by enhancing ferroptosis both in vitro and in vivo.^111^ Ku et al. revealed that **furamidine (FM)** inhibited PRMT1, reducing the H4R3me2 modification, chromatin accessibility, and expression of glycolysis-related genes to reverse the chemoresistance of pancreatic cancer.^387^ Treated the CRC organoids by the histone demethylase inhibitor, **methylstat**, significantly restore the H4K20me3 and H3K27me3 levels in a dose-dependent manner, meanwhile reduced organoids growth.^104^

### Therapeutic opportunity by targeting TF or co-TF

The transcription and cotranscription factors (TFs and co-TFs) can occupy the cis-acting element specifically to determine the local accessibility of chromatin. Thus, targeting TF and co-TF is promising strategies to remodel chromatin accessibility.

All intracellular receptors are trans-acting factors, such as the androgen receptor (AR). AR inhibitors are applied for the treatment of prostate cancer, but drug resistance is a major clinical problem. Leppänen et al. revealed that the AR inhibitor, **enzalutamide (ENZ)**, increased the chromatin accessibility and expression of SIX2, facilitating stem-like reprogramming, neuroendocrine differentiation, and AR inhibitors resistance.^554^ ONECUT2 was a prostate cancer trigger through remodel the chromatin accessibility landscape independent of AR. The activation of ONECUT2 was an novel mechanisms of drug resistance, and inhibiting ONECUT2 by **CSRM-617** reshaped chromatin accessibility, expression profile, and cell lineage of prostate cancer cells.^555^ **MYCi975** is a Myc inhibitor that disrupts Myc/Max dimerization.^556^ Holmes et al. demonstrated that **MYCi975** reduced H3K27ac and chromatin accessibility at the binding motifs of CTCF and FOX family transcription factors.^557^ **AZD4573**, an CDK9 inhibitor, remodeled the global promoter and enhancer accessibility as well as inhibited the proliferation and induced the apoptosis of lymphoma cells.^558^ Seredinski et al. indicated that the **acriflavine**, inhibited DNA topoisomerase, increasing the locus accessibility and expression of many lncRNAs to suppress the growth of endothelial cells.^559^ Additionally, other selective inhibitor also attenuated the recruitment of TFs to their binding motifs, such as **HLM006474** (E2F inhibitor), **S3I-201** (STAT3 inhibitor),^560^ **Am80** (KLF5 inhibitor),^561^ **gliotoxin** (NOTCH2 inhibitor),^562^ and so on.

Besides, targeting the specific kinases of the TFs also is a viable strategy for chromatin remodeling, such as **XAV-939** (TNKS1/2 inhibitor) and **VT02956** (LATS inhibitor) to determine WNT/β-catenin- and Hippo/YAP-mediated chromatin dynamics, respectively.^563,564^

As mentioned above, there are many targeting compounds to remodel chromatin structure, however, most of them have not yet entered clinical or preclinical trials. Even if some inhibitors have got clinical trials, the therapeutic effect are still not satisfactory. The main reasons include the low specificity and bioavailability of these inhibitors. In addition, most of the targets are multifunctional and existed in different complexes, therefore, simply incapacitating these targets may cause a variety of functional disturbances leading to serious side effects. Hence, in parallel with searching for more selective inhibitors, screening out inhibitors to specifically target the assembly of specific complexes may be a more promising strategy to reverse the dysregulation chromatin accessibility.

## Conclusion and perspective

The conformity of structure and function is one of the basic characteristics of living organisms. The hereditary information of eukaryotes is contained in chromosomes, which contain DNA, histones, non-histone proteins, and a little RNA, forming a multilevel structure encapsulated in the nucleus. Nucleosomes, the basic structure of chromatin, are highly enriched in inaccessible regions, but relatively reduced in open regions, which correspond with their transcription potential, DNA replication, and repair capacity.

The chromatin accessibility is dynamically regulated. Nucleosome localization and occupancy are the most dominated parameters of chromatin accessibility. The open portion of chromatin is not completely free DNA, but dynamically occupied by histones and other DNA-binding factors which regulate the opening state of chromatin. Additionally, the RNA polymerase can relocate nucleosomes during transcription elongation, rather than completely expel them, hinting that dynamically occupied by nucleosomes, but free DNA is required for gene transcription.^565^ Besides, chromatin openness and local transcriptional activity are not completely consistent. For example, nucleosomes, located at the TSS, usually inhibit transcription, but can also promote RNA polymerase entry when the nucleosomes are located appropriately.^566^ Recent research indicated that the nucleosomes located in TSS are essential for transcription by recruiting the transcription preinitiation complex.^567^ Some enhancer and promoter regions are accessible but showed low transcription activity for the lack of active modification markers, suggesting that the advanced topological structure and modification of local chromatin are also required for transcription.^568^ In addition, many epigenetic modifications are believed to indicate chromatin accessibility, but it’s also not absolute corresponded.^328^ Hence, the code of chromatin accessibility, nucleosome occupancy, epigenetic modification, and transcription activity requires further research to decode.

Chromatin accessibility participates in many cellular progresses, such as alternative splicing, DNA repair, immune and inflammatory responses, which are widely involved in embryogenesis, neurodegenerative disease, cardiovascular disease, diabetes, COVID-19 infection, and so on. Changes in chromatin accessibility and gene transcription are sequential, and the variation of chromatin accessibility always precede that of transcription.^347^ Hence, chromatin accessibility based information can raise an earlier prediction for the initiation, progression, and prognosis of many diseases. Up to now, several chromatin accessibility-based strategies have been applied in the clinical or preclinical diagnosis and treatment of different diseases. Many disease susceptibility risk loci are screened out by genome-wide association study, combined with high-throughput chromatin accessibility detection, we can comprehensively analyze the correlation between diseases and the accessibility variation at specific sites. CRISPR has great advantages in specific site editing, and CRISPR-based therapeutics have been used for the treatment of some diseases.^569^ The existing CRISPR-based treatment is mainly focused on DNA editing. Currently, scientists have achieved CRISPR-based epigenetic editing to control gene expression, having a much lower genomic mutation risk, which has a greater advantage in therapeutic application.^570^ Additionally, given the specificity of TFs to their binding motifs, they also are ideal candidate targets for site-specific epigenetic editing. Similar to PROTAC, a chemical inducer assembled Bcl6 with CDK9, phosphorylating RNA polymerases to promote the transcription of specific genes.^571^ It suggests that we can apply chemical inducer of proximity that can assemble the protein complexes with transcription factor to regulate the local chromatin accessibility. This strategy allow us to utilize the “enemy” (disease inducer) to defeat the “enemy” (disease) through precision striking. Besides, non-coding RNA drugs have become an important research direction for targeted therapy.^572^ Considering the role of non-coding RNAs in chromatin accessibility regulation, targeting non-coding RNA-mediated chromatin remodeling could be a promising field.

The laboratory results are always encouraging, however, the outcomes of clinical application are more about unsatisfactory, and the main reasons at least include the limitations in specificity and bioavailability. Fortunately, the high-throughput drug screening strategies allow us to obtain potential drugs more effectively. Meanwhile, the experimental model that can better imitate the real situation of patients, such as organoids, could further enhance the clinical application prospects of various drugs. Besides, the organism-wide chromatin accessibility profiles can pinpoint the regulatory variants during the development and disease progress, helping to propose more precise treatment strategies. In summary, yet there are many challenges, it’s sure that the investigation on chromatin accessibility will bring more and more help and surprise to our life science research and disease treatment.

## Data Availability

All the data of this study were available from the corresponding authors on reasonable request.
